# Mass HIV Treatment and Sex Disparities in Life Expectancy: Demographic Surveillance in Rural South Africa

**DOI:** 10.1371/journal.pmed.1001905

**Published:** 2015-11-24

**Authors:** Jacob Bor, Sydney Rosen, Natsayi Chimbindi, Noah Haber, Kobus Herbst, Tinofa Mutevedzi, Frank Tanser, Deenan Pillay, Till Bärnighausen

**Affiliations:** 1 Department of Global Health, Boston University School of Public Health, Boston, Massachusetts, United States of America; 2 Africa Centre for Population Health, Mtubatuba, South Africa; 3 Health Economics and Epidemiology Research Office, Department of Internal Medicine, School of Clinical Medicine, Faculty of Health Sciences, University of Witwatersrand, Johannesburg, South Africa; 4 Department of Global Health and Population, Harvard T. H. Chan School of Public Health, Boston, Massachusetts, United States of America; 5 Faculty of Medical Sciences, University College London, London, United Kingdom; Massachusetts General Hospital, UNITED STATES

## Abstract

**Background:**

Women have better patient outcomes in HIV care and treatment than men in sub-Saharan Africa. We assessed—at the population level—whether and to what extent mass HIV treatment is associated with changes in sex disparities in adult life expectancy, a summary metric of survival capturing mortality across the full cascade of HIV care. We also determined sex-specific trends in HIV mortality and the distribution of HIV-related deaths in men and women prior to and at each stage of the clinical cascade.

**Methods and Findings:**

Data were collected on all deaths occurring from 2001 to 2011 in a large population-based surveillance cohort (52,964 women and 45,688 men, ages 15 y and older) in rural KwaZulu-Natal, South Africa. Cause of death was ascertained by verbal autopsy (93% response rate). Demographic data were linked at the individual level to clinical records from the public sector HIV treatment and care program that serves the region. Annual rates of HIV-related mortality were assessed for men and women separately, and female-to-male rate ratios were estimated in exponential hazard models. Sex-specific trends in adult life expectancy and HIV-cause-deleted adult life expectancy were calculated. The proportions of HIV deaths that accrued to men and women at different stages in the HIV cascade of care were estimated annually.

Following the beginning of HIV treatment scale-up in 2004, HIV mortality declined among both men and women. Female adult life expectancy increased from 51.3 y (95% CI 49.7, 52.8) in 2003 to 64.5 y (95% CI 62.7, 66.4) in 2011, a gain of 13.2 y. Male adult life expectancy increased from 46.9 y (95% CI 45.6, 48.2) in 2003 to 55.9 y (95% CI 54.3, 57.5) in 2011, a gain of 9.0 y. The gap between female and male adult life expectancy doubled, from 4.4 y in 2003 to 8.6 y in 2011, a difference of 4.3 y (95% CI 0.9, 7.6). For women, HIV mortality declined from 1.60 deaths per 100 person-years (95% CI 1.46, 1.75) in 2003 to 0.56 per 100 person-years (95% CI 0.48, 0.65) in 2011. For men, HIV-related mortality declined from 1.71 per 100 person-years (95% CI 1.55, 1.88) to 0.76 per 100 person-years (95% CI 0.67, 0.87) in the same period. The female-to-male rate ratio for HIV mortality declined from 0.93 (95% CI 0.82–1.07) in 2003 to 0.73 (95% CI 0.60–0.89) in 2011, a statistically significant decline (*p =* 0.046). In 2011, 57% and 41% of HIV-related deaths occurred among men and women, respectively, who had never sought care for HIV in spite of the widespread availability of free HIV treatment. The results presented here come from a poor rural setting in southern Africa with high HIV prevalence and high HIV treatment coverage; broader generalizability is unknown. Additionally, factors other than HIV treatment scale-up may have influenced population mortality trends.

**Conclusions:**

Mass HIV treatment has been accompanied by faster declines in HIV mortality among women than men and a growing female–male disparity in adult life expectancy at the population level. In 2011, over half of male HIV deaths occurred in men who had never sought clinical HIV care. Interventions to increase HIV testing and linkage to care among men are urgently needed.

## Abstract 2

Jacob Bor and colleagues use demographic data from a longitudinal surveillance cohort to identify increased gains in life expectancy among women compared to men in the years following antiretroviral therapy scale-up in rural South Africa.

## Editors' Summary

**Background.:**

AIDS has killed 39 million people over the past three decades, and about 35 million people (including 25 million living in sub-Saharan Africa) are currently infected with HIV, the retrovirus that causes AIDS. HIV destroys immune system cells, leaving HIV-positive individuals susceptible to other serious infections. Early in the AIDS epidemic, most HIV-positive individuals died within ten years of infection. Then, in 1996, effective antiretroviral therapy (ART) became available. For people living in high-income countries, HIV infection became a chronic condition, but HIV/AIDS remained largely untreated and fatal in resource-limited countries. In 2003, the international community began to work towards achieving universal access to ART. Now, at least a third of people living with HIV have access to ART, the global rate of AIDS-related deaths has fallen by more than a third from its 2005 peak, and the life expectancy (how long a person is likely to live based on their year of their birth, their current age, and other demographic factors) of HIV-positive adults has markedly increased.

**Why Was This Study Done?:**

Although ART has been provided without charge to patients in South African public clinics since 2004, HIV/AIDS remains the leading cause of death in many parts of South Africa. Reducing the lingering burden of HIV mortality is a policy priority. Prior studies have found worse outcomes among men in HIV care and treatment. Here, the researchers assess the evolution of sex disparities in life expectancy and HIV mortality rates at the population level with the scale-up of ART in a rural region of KwaZulu-Natal, South Africa, where 29% of the population is HIV-positive. Different trends in life expectancy for men and women following ART scale-up may reflect differences in the underlying burden of HIV disease—women tend to be infected and die at younger ages—as well as differences in access to HIV testing, care, and treatment. The researchers also assess where in the cascade of HIV care and treatment HIV mortality occurs for men and women. Knowing more about sex-specific trends in population life expectancy and where the lingering burden of HIV mortality occurs could help experts design strategies to further reduce HIV mortality in regions where free ART is widely available.

**What Did the Researchers Do and Find?:**

The researchers linked demographic data obtained through regular household surveys, including all deaths occurring between 2001 and 2011, for nearly 100,000 adults living in rural KwaZulu-Natal to clinical records held by the public sector HIV treatment and care program. In addition, trained nurses visited households where there had been a death and collected data that were used to determine the probable cause of death (verbal autopsy). Analysis of these data indicated that, between 2003 and 2011, female adult life expectancy increased by 13.2 years (from 51.3 years to 64.5 years), whereas male life expectancy increased by only 9 years (from 46.9 years to 55.9 years). Thus, the gap between female and male adult life expectancy doubled between 2003 and 2011. Moreover, although HIV-related mortality among women and men was similar in 2003, in 2011 women were 27% less likely to die from HIV than men. With the scale-up of ART, male sex has emerged as a risk factor for HIV mortality at the population level. Finally, in 2011, 55% of all male HIV-related deaths and 40% of all female HIV-related deaths occurred among men and women who had never sought care for HIV/AIDS.

**What Do These Findings Mean?:**

These findings suggest that although the mass provision of free ART in South Africa has coincided with a reduction in HIV-related mortality among both men and women, ART scale-up has been accompanied by faster declines in HIV-related deaths among women than men and by a growing female–male disparity in adult life expectancy. Notably, these findings also indicate that despite the wide availability of ART, about half of all HIV-related deaths in the study population occurred among people who had never sought care for HIV. The use of verbal autopsy to determine the probable cause of death may limit the accuracy of these findings, and factors other than the scale-up of ART may have influenced the population mortality trends. Moreover, these findings may not be generalizable to other populations. However, these results highlight the need for further research to understand why men are not as likely as women to seek and adhere to HIV care and treatment, and to design effective interventions to increase the uptake of HIV services among men. Without better outreach to men, the researchers conclude, the full benefits of mass ART provision will not be realized.

**Additional Information.:**

This list of resources contains links that can be accessed when viewing the PDF on a device or via the online version of the article at http://dx.doi.org/10.1371/journal.pmed.1001905.

- Information is available from the US National Institute of Allergy and Infectious Diseases on HIV infection and AIDS
- NAM/aidsmap provides basic information about HIV/AIDS, summaries of recent research findings on HIV care and treatment, and personal stories about living with HIV/AIDS
- Information is available from Avert, an international AIDS charity, on many aspects of HIV/AIDS, including information on universal access to ART and on HIV/AIDS in South Africa; Avert also provides personal stories about living with HIV/AIDS
- The World Health Organization (WHO) provides information on all aspects of HIV/AIDS (in several languages), including its *Consolidated Guidelines on the Use of Antiretroviral Therapy for Treating and Preventing HIV Infection* and information on the WHO/UNAIDS Treatment 2.0 strategy, an initiative to expand access to HIV testing and ART
- The UNAIDS Fast-Track Strategy to End the AIDS Epidemic by 2030 provides up-to-date information about the AIDS epidemic and efforts to halt it, including progress towards universal access to antiretroviral therapy; UNAIDS also provides detailed information about HIV/AIDS in South Africa
- More information about the population-based surveillance cohort that provided the data for this study is available from the Africa Centre for Population Health website

## Introduction

The scale-up of HIV antiretroviral therapy (ART) in HIV-endemic settings has led to large gains in adult life expectancy at the population level [1,2]. Yet in many parts of southern Africa, HIV remains the leading cause of death [3]. Since 2004, South Africa has provided ART at no charge to patients in public sector clinics and hospitals, and over 2 million South Africans are currently receiving therapy [4].

This paper investigates the differential impact of mass HIV treatment on survival of men and women in the general population. We assess sex-specific trends in adult life expectancy and HIV-related mortality. To shed light on why men and women continue to die from HIV when ART is widely available, we investigate where in the cascade of care HIV mortality occurs, how these patterns differ by sex, and how they have changed over time with ART scale-up.

Globally, female life expectancy is about 4.7 y longer than male life expectancy (70.9 versus 66.2 y) [5], yet this life expectancy gap varies across space and time. Female–male differences in life expectancy are the product of biological, behavioral, and environmental factors, including sex-specific patterns in exposure to disease risks and access to treatments. During the 1990s and early 2000s, epidemic HIV/AIDS compressed the female–male life expectancy gap in many of the hardest hit countries in sub-Saharan Africa (S1 Fig) [5]. Little is known about how the expansion of HIV treatment has affected disparities in sex-specific survival.

Differential trends in life expectancy for men and women with ART scale-up may reflect both differences in the underlying burden of HIV disease and differences in access to HIV care and treatment services. Previous studies in southern Africa have documented sex differences in rates of progression at specific stages in the HIV cascade of care. Women have higher rates of HIV testing [6] and linkage to care [7–9] than men. Among patients not yet eligible for ART, women are more likely to be retained in pre-ART care [10]. Men tend to initiate ART at lower CD4 counts and in worse health [11–13]. And there is evidence that men have worse adherence [14] and lower retention on therapy [12] once initiated on ART. Sex differences have also been widely reported in health outcomes among patients who have initiated ART, with male patients experiencing higher mortality [12,15–17] and worse recovery of immune function [17].

The cumulative impact of these relative disadvantages for men and how they have evolved over time has not been described. We assess changes in survival among men and women ≥15 y in the general population in an entire community under continuous demographic and health surveillance before and during the rapid scale-up of ART. This community is characterized by very high HIV prevalence [18] and high ART coverage [1,19]. By taking a population-based perspective, we capture the full cascade of HIV care and treatment, including persons who have not yet accessed the medical system. This is critically important given the high proportion of HIV-infected South Africans who are not aware that they are HIV-positive (62% of males and 45% of females in 2012) [6] and who nevertheless may be at risk for death due to HIV. Facility-based studies do not observe this important population, and cross-sectional HIV surveillance studies do not measure mortality. The analysis presented here highlights opportunities for future interventions to reduce the lingering burden of HIV mortality in the context of nominally free and widely available treatment.

## Methods

### Ethics

Ethical approval for data collection, linkage, and analysis by the Africa Centre for Population Health (Africa Centre) was obtained from the University of KwaZulu-Natal Biomedical Research Ethics Committee. Verbal informed consent was obtained from household respondents. The analyses conducted for this paper consisted of secondary analysis of de-identified data.

### Data

The Africa Centre is one of the Wellcome Trust's five major overseas programs. As one of its core activities, the Africa Centre operates a large health and demographic surveillance site in rural KwaZulu-Natal, South Africa. All members of all households located in a 438-km^2^ demographic surveillance area (DSA) are followed up longitudinally on a range of demographic, health, and socioeconomic indicators through regular household surveys. The area has an adult HIV prevalence of 29% [18], and just 34% of working age adults are employed [20]. Since 2000, the Africa Centre has conducted demographic surveillance through semi-annual household visits. Over 150,000 individuals in more than 20,000 households have been included in the surveillance; household response rates exceed 99% [21]. The surveillance population includes all members of all households—including household members who are not currently residing in the surveillance area (non-resident household members), which is an important feature given high rates of migration in rural South Africa. Following every death in the surveillance area that is recorded by the surveillance teams, the household of the deceased is visited by a trained nurse who conducts a verbal autopsy interview to determine the probable cause of death. Data on the events leading up to the death are recorded in a standardized form; these data are fed into a computerized algorithm (InterVA), and a cause of death is probabilistically assigned [22,23]. Response rates in the verbal autopsy data are very high. During the period 2001–2011, 93.0% of all deaths had a cause of death assigned by verbal autopsy, 5.3% were missing/refused, and 1.7% were indeterminate.

In addition to the demographic surveillance, the Africa Centre maintains a clinical database for all patients in the public sector HIV and Treatment program (Hlabisa HIV Treatment and Care Programme) that has served the DSA since the national HIV treatment program began in 2004. Clinical records have been linked at the individual level with the demographic surveillance using national identification numbers, full name, age, and sex, enabling assessment of the demographic implications of public sector treatment scale-up [19,20]. Private sector utilization for ART is low in the community due to the high cost of ART outside the public sector and low levels of private insurance coverage.

For this analysis, dates of birth, death, and residency episodes were obtained for the complete population under surveillance from 1 January 2001 through 31 December 2011. Deaths were coded as HIV-related or not HIV-related based on the verbal autopsy interviews. Since tuberculosis (TB) is a common opportunistic infection and common immediate cause of death for people with HIV in this community, TB-related deaths were coded as HIV-related, consistent with previous analyses of these data [1,22,24,25]. The use of verbal autopsy to identify HIV-related deaths has important advantages over approaches using HIV biomarker surveillance, an alternate approach in the literature [2]. First, deaths occurring in HIV-infected persons but due to other causes (e.g., vehicle accidents, non-communicable diseases) are not attributed to HIV. Second, our approach is not vulnerable to the high rates of non-response in HIV biomarker surveillance, which is likely to be correlated with true HIV status [26].

Into the Africa Centre’s demographic database were merged clinical data from the public sector HIV care and treatment program: date of first recorded CD4 count—a proxy for the date of entry into clinical HIV care—and date of ART initiation. We divided person-time into four mutually exclusive, collectively exhaustive categories: (1) never sought care (no CD4 count); (2) sought care (CD4 count) but never initiated ART; (3) initiated ART within the previous year; (4) initiated ART more than 1 y ago. We distinguish between the first and later years on ART due to widely documented higher mortality in the first year on therapy [27].

### Study Population and Inclusion Criteria

The study population included all resident and non-resident adult (≥15 y old) members of all households under surveillance, 2001–2011. Dates and causes of death were ascertained through household proxy for all members of the study population—regardless of whether they resided in the surveillance area. Person-time was included from the date an individual was first observed in the surveillance or their 15th birthday until the date that person exited the surveillance, through death or because they ceased to be a member of a household under surveillance. For analyses that disaggregated individuals by whether they had sought care or initiated ART, we limited the study population to persons residing in the DSA for at least 90 days, because non-resident household members would have been more likely to seek HIV care and treatment outside the Hlabisa catchment area.

### Data Analysis

#### Sex-specific trends in adult life expectancy

Life expectancy is a summary metric of the mortality experience in a population and is widely used to compare mortality across populations and over time. Life expectancy is an expectation in the statistical sense: it is the mean number of years a cohort would live if it were exposed to the full profile of age-specific all-cause mortality rates observed in a population in a given period of time. Life expectancy is a period measure and may differ from the average length of life for members of a birth cohort, which is only observed after all members have died. For example, if age-specific mortality rates declined with calendar time, then life expectancy would underestimate the average length of life for a birth cohort.

Adult life expectancy is the number of additional years of life expected, conditional on survival to age 15 y, and is commonly denoted *e*_15_. We calculated *e*_15_ separately for men and women using a continuous-time approach[1]: for each calendar year in 2001–2011, we estimated sex-specific survival curves beginning at age 15 y, using the non-parametric Kaplan-Meier estimator. To obtain annual sex-specific estimates of *e*_15_, we numerically integrated under the annual sex-specific survival curves. Due to sparseness of data beyond age 95 y, we censored the annual survival curves at age 95 y and calculated the expected number of years lived in the 80-y interval between ages 15 and 95 y (_80_ *e*_15_). A correction factor equal to life expectancy at age 95 y (e_95_) was estimated separately for each sex for the entire period 2001–2011 and was added to our annual estimates of _80_ *e*_15_ to obtain annual estimates of *e*_15_. Throughout this paper, we report adult life expectancy as *e*_15_ + 15 y, so that estimates correspond to ages at death rather than years remaining at age 15 y [1]. To quantify the evolution of sex disparities in adult life expectancy, we calculated the female-to-male gap in adult life expectancy in each calendar year for 2001–2011, sex-specific changes in adult life expectancy between 2003—prior to the beginning of ART scale-up—and 2011, and the difference in sex-specific changes in adult life expectancy from 2003 to 2011. We bootstrapped at the individual level (101 resamples) to estimate standard errors and constructed 95% percent confidence intervals for each of these comparisons.

To assess the contribution of HIV-related mortality to trends in adult life expectancy, we next estimated HIV-cause-deleted adult life expectancy, separately for men and women. HIV-cause-deleted adult life expectancy was estimated using the approach described above, except that HIV-related deaths identified in verbal autopsy were coded as censored on the date of death rather than included as deaths. Mechanistically, trends in HIV-cause-deleted life expectancy show changes in the rate and age distribution of mortality due to all other causes. Under the assumption that mortality due to HIV and mortality due to other causes are independent, HIV-cause-deleted life expectancy reflects the life expectancy that would exist if HIV-related mortality were eliminated. Under this assumption, the difference between HIV-cause-deleted adult life expectancy and adult life expectancy would reflect the years of life lost due to HIV. Although the independence assumption cannot be tested directly, it is consistent with prior evidence that HIV-infected persons on ART in sub-Saharan Africa have life expectancies close to those in the general population [27,28]. Sensitivity analyses assessing the robustness of our HIV-cause-deleted life expectancy estimates to deviations from the independence assumption—as well as further discussion of this assumption—are presented in S1 Text.

#### Sex-specific trends in HIV-related mortality

We assessed trends in HIV-specific mortality rates by sex and estimated trends in female-to-male HIV mortality rate ratios. If ART scale-up had the same proportional effect on HIV mortality for men and women, then the ratio of HIV mortality for women to HIV mortality for men would be expected to be constant over time. We estimated female-to-male HIV mortality rate ratios for each calendar year in 2001–2011 using an exponential hazard model, in which time to HIV death was regressed on indicators for each calendar year and the interaction of those indicators with sex. To test whether the relative change in HIV mortality over time differed for men and women, we estimated a similar hazard model but included a main effect for female, set calendar year = 2003 as the reference category, and made inferences on the interaction between female and calendar year = 2011. To control for potential confounding by age, we additionally controlled for age group indicators (15–29, 30–44, 45–64, 65+ y) in an age-adjusted hazard model. Finally, we stratified the analysis by age. We regressed time to HIV death on indicators for year-by-age strata and indicators for sex-by-year-by-age strata. Annual age-specific female-to-male HIV mortality rate ratios were obtained by exponentiating the coefficients on the latter terms, and we tested the null hypothesis that the relative changes in HIV mortality for women versus men over the period 2003–2011 were constant across age groups. In all models, person-time was censored at exit from the surveillance, either because a person ceased to be a member of a household under surveillance or because he or she died from another cause. To adjust for non-independence of the person-time contributed by the same individuals in different years, we clustered standard errors at the individual level in all regression analyses.

#### Sex-specific differences in progression through the HIV cascade of care and attribution of HIV-related deaths across the cascade

Differences in HIV mortality trends may be due to differences in access to HIV care and treatment. To assess sex differences in progression through the HIV cascade of care, we calculated the proportion of all surviving adults (ages 15 y and over) in the population residing in the DSA who (1) had ever sought care in the public sector ART program, as evidenced by a recorded CD4 count, or (2) had ever initiated ART in the public sector ART program. Proportions were assessed annually for 2001–2011 at mid-year (July 1), and exact 95% CIs estimated.

We disaggregated HIV-related deaths into four groups defined by whether the deceased had ever sought care and/or initiated ART in the public sector ART program: (1) never sought care. (2) sought care but never initiated ART, (3) initiated ART less than 1 y prior to death, or (4) initiated ART more than 1 y prior to death. This analysis was limited to persons who were at least 15 y old on their date of death. We also excluded persons who migrated into the DSA in the 90 d before their date of death, in order to exclude people who died from HIV but might not have had the opportunity to seek care for HIV in the local public sector ART program [29]. For each calendar year from 2001 to 2011, the distribution of deaths across these four care-seeking categories was evaluated separately for men and women. Lastly, HIV mortality rates were calculated for the population residing in the DSA on 1 January 2011, by care-seeking category. Female-to-male HIV mortality rate ratios were estimated separately for each stage in exponential hazard models adjusting for age (15–29, 30–44, 45–64, 65+ y). In alternate specifications, we additionally controlled for log(CD4 count at entry into care + 1) for stage 2 above and for log(last CD4 count before ART initiation + 1) for stages 3 and 4 above. Person-time was censored at exit from the surveillance, death due to a competing cause, or the date of transition to the next stage in the cascade of care.

The validity of our attribution of HIV-related deaths across the cascade of care depends on the accuracy of verbal autopsy coding. Persons who died from other causes but whose death was attributed to HIV might not have even been HIV positive and would have been less likely to have sought care for HIV since they would have had no reason to do so. In robustness checks, we limited the sample of deaths to those that specifically named HIV as a cause, those that named HIV as a cause with an InterVA likelihood score > 90%, those that named either HIV or TB as a cause, and those that named either HIV or TB as a cause with an InterVA likelihood score > 90%. For further information, see S2 and S3 Texts.

## Results

All adult person-time contributed by members of the demographic surveillance from 2001 through 2011 was analyzed. In all, 52,964 women and 45,688 men contributed a total of 615,075 person-years to the analysis (Table 1). Attrition from the population surveillance was comparable for men and women, at 3.0 and 3.3 per 100 person-years, respectively. A total of 12,290 deaths were reported during follow-up, of which 7,229 (58.8%) were identified by verbal autopsy to be HIV/TB-related.

**Table 1:** Population demographic surveillance 2001–2011: summary statistics.

Summary Statistic	Women	Men
Individuals	52,964	45,688
Person-years	331,476	283,599
Deaths	6,140	6,150
Mortality rate (per 100 PY)	1.85	2.17
HIV/TB-related deaths	3,729	3,500
HIV/TB mortality rate (per 100 PY)	1.12	1.23
Persons lost from surveillance	10,976	8,529
Attrition rate (per 100 PY)	3.31	3.01

Study population includes all members of all households in the DSA over age 15 y. Individuals come under observation when they turn 15 y old or when they join a household under surveillance. Individuals exit observation when they cease to be members of a household under surveillance. Non-resident household members were included in the assessment of mortality trends but were excluded from the analysis decomposing HIV deaths across the cascade of care. PY, person-years.

**Fig 1:**
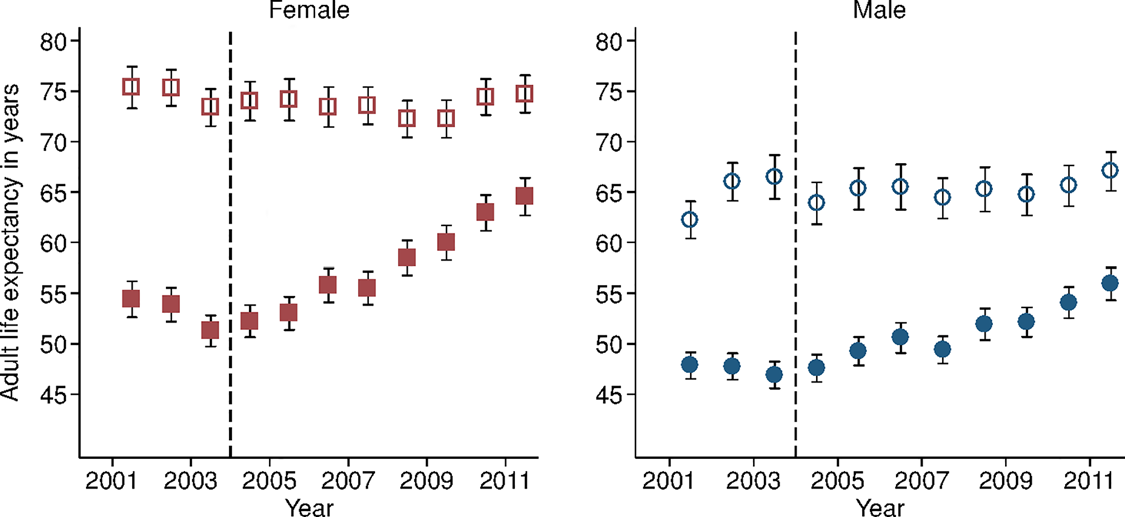
Adult life expectancy and HIV-cause-deleted adult life expectancy, 2001–2011, by sex. Solid symbols are annual estimates of adult life expectancy; open symbols are annual estimates of HIV-cause-deleted adult life expectancy. 95% CIs are shown. The black dashed line indicates the beginning of ART scale-up in 2004.

**Fig 2:**
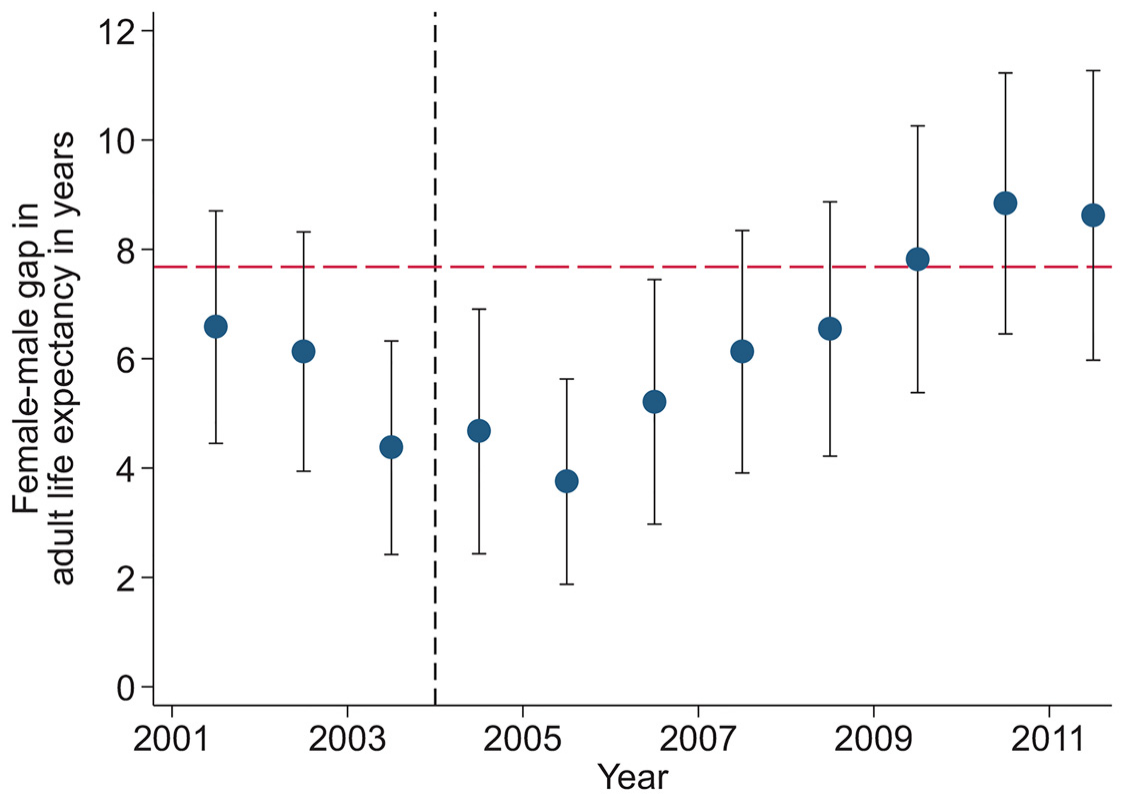
Female–male difference in adult life expectancy, 2001–2011. Solid blue circles display annual estimates of the gap between female and male adult life expectancy. The red dashed line indicates the gap in HIV-cause-deleted life expectancy observed in 2011. The black dashed line indicates the beginning of ART scale-up in 2004.

Sex-specific trends in adult life expectancy are presented in Fig 1. For both men and women, adult life expectancy fell in the early 2000s and then began to rise after ART scale-up began in 2004. Women experienced substantially larger gains in survival than men, however. For women, adult life expectancy increased from 51.3 y (95% CI 49.7, 52.8) in 2003 to 64.5 y (95% CI 62.7, 66.4) in 2011, a gain of 13.2 y 95% CI 10.7, 15.8). For men, adult life expectancy increased from 46.9 y (95% CI 45.6, 48.2) in 2003 to 55.9 y (95% CI 54.3, 57.5) in 2011, a gain of 9.0 y (95% CI 6.9, 11.1). Over this period, the female–male gap in adult life expectancy nearly doubled, from 4.4 y in 2003 (95% CI 2.3, 6.5) to 8.6 y in 2011 (95% CI 6.1, 11.1) (Fig 2), a statistically significant difference of 4.3 y (95% CI 0.9, 7.6).

**Fig 3:**
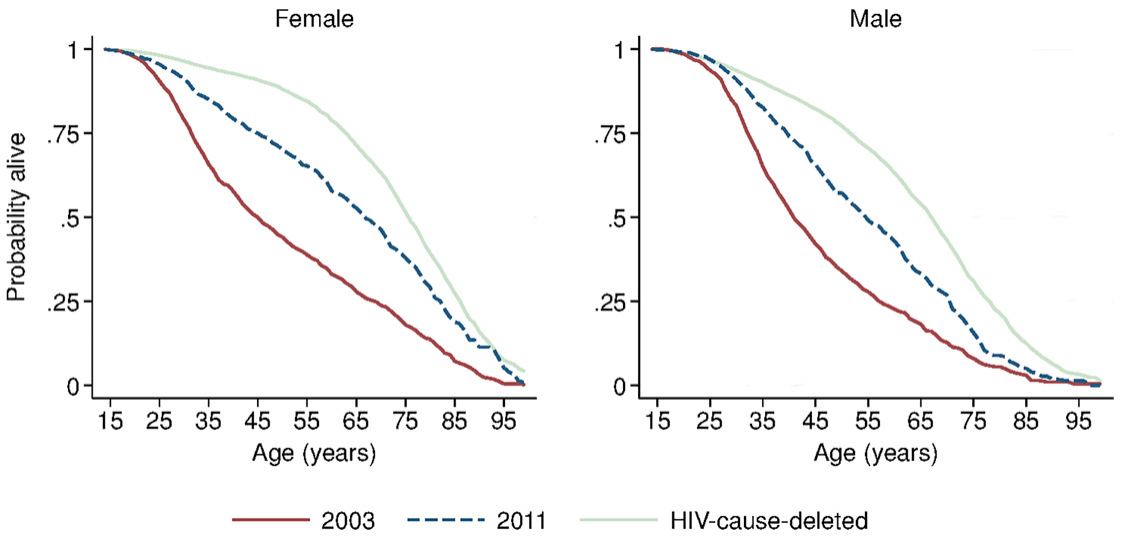
Sex-specific survival curves: 2003, 2011, and HIV-cause-deleted. Sex-specific continuous-time Kaplan-Meier survival curves for 2003 and 2011 are shown. The HIV-cause-deleted survival curves pool person-time for 2001–2011. These are period (synthetic cohort) survival curves reflecting age-specific mortality rates in a population in a given period of time; life expectancy is calculated as the area under the curve. A risk table showing persons at risk and deaths at each age for each of the six survival curves is available as S1 Table.

During the period 2001–2011, HIV-cause-deleted life expectancy remained approximately stable in both men and women (Fig 1), indicating that the changes in adult life expectancy were indeed driven by changes in HIV-related mortality. As Fig 1 shows, however, HIV-cause-deleted life expectancy is much higher for women than for men at about 75 and 65 y, respectively, largely because of the high burden of mortality from injury for young men in this setting [30]. Because of lower competing mortality risks, women have larger potential gains in years of life from ART scale-up; in other words, the benefits of avoiding HIV mortality are greater for women than for men. The “potential gains” in survival can be seen in the outermost curves in Fig 3, which compares sex-specific survival curves for 2003, 2011, and HIV-cause-deleted survival. (S1 Table presents the risk table corresponding to Fig 3.) However, as shown in Fig 3, not only do women have longer potential life expectancy: by 2011, women were also relatively closer than men to recovering the HIV-cause-deleted estimate of their potential life expectancy, vis-à-vis the 2003 nadir in life expectancy prior to the public sector scale-up of ART. Interpreting HIV-cause-deleted life expectancy as the potential life expectancy that could be achieved in the absence of HIV-related mortality depends on the assumption that HIV and other causes of death are independent. In sensitivity analyses, in which we computed alternate estimates of HIV-cause-deleted adult life expectancy assuming very strong positive (or negative) dependence between HIV and other causes of death, our results were not substantially changed (S1 Text).

Table 2 presents population HIV mortality rates for men and women for each calendar year in 2001–2011, as well as female-to-male HIV mortality rate ratios. HIV mortality declined in both men and women, but declined faster for women. Among women, HIV mortality declined from 1.60 deaths per 100 person-years (95% CI 1.46, 1.75) in 2003 to 0.56 per 100 person-years (95% CI 0.48, 0.65) in 2011. For men, HIV-related mortality declined from 1.71 per 100 person-years (95% CI 1.55, 1.88) to 0.76 per 100 person-years (95% CI 0.67, 0.87) over the same period. Whereas women were no less likely than men to die from HIV in 2003 (rate ratio [RR] = 0.93; 95% CI 0.82, 1.07), by 2011 women were 27% less likely to die from HIV than men (RR = 0.73; 95% CI 0.60, 0.89). The decline in the relative rate of HIV death for women vis-à-vis men from 2003 to 2011 was statistically significant in both the crude (RR = 0.78; 95% CI 0.61, 1.00; *p =* 0.046) and age-adjusted analysis (RR = 0.77; 95% CI 0.61, 0.98; *p =* 0.036) (S2 Table).

**Table 2:** HIV mortality rates for females and males, 2001–2011.

Year	Female			Male			Female-to-Male Rate Ratio (95% CI)
	Deaths	PY/100	Rate	Deaths	PY/100	Rate	
2001	379	278.3	1.36	347	237.6	1.46	0.93 (0.81, 1.08)
2002	413	288.4	1.43	403	245.6	1.64	0.87 (0.76, 1.00)
2003	465	291.0	1.60	423	247.4	1.71	0.93 (0.82, 1.07)
2004	440	293.9	1.50	371	249.7	1.49	1.01 (0.88, 1.16)
2005	421	296.6	1.42	341	252.2	1.35	1.05 (0.91, 1.21)
2006	354	301.0	1.18	301	256.7	1.17	1.00 (0.86, 1.17)
2007	369	308.2	1.20	339	263.7	1.29	0.93 (0.80, 1.08)
2008	264	309.9	0.85	268	265.9	1.01	0.85 (0.71, 1.00)
2009	235	312.0	0.75	263	268.4	0.98	0.77 (0.64, 0.92)
2010	211	316.6	0.67	234	273.5	0.86	0.78 (0.65, 0.94)
2011	178	318.9	0.56	210	275.4	0.76	0.73 (0.60, 0.89)

PY/100 = person-years divided by 100. The table includes all adult (15+ y) members of households in the demographic surveillance. Female-to-male rate ratios were estimated in an exponential hazard regression model in which time to HIV-related death was regressed on calendar year and the interaction between calendar year and female.

**Fig 4:**
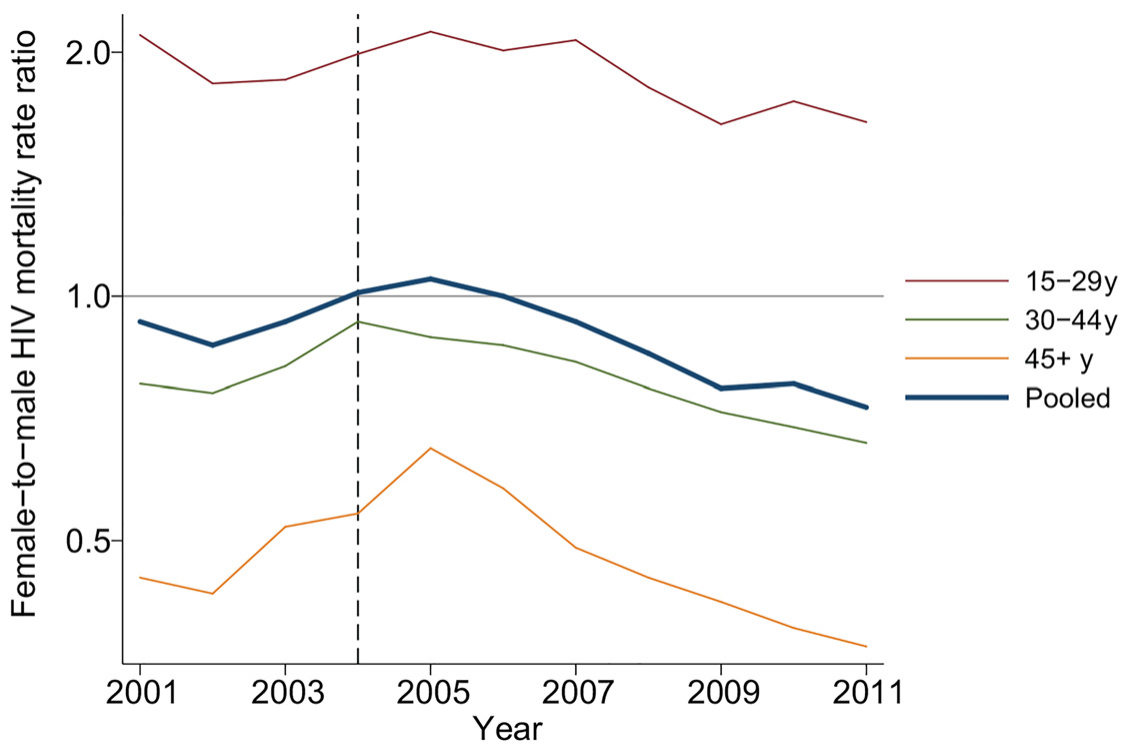
Female-to-male HIV mortality rate ratios by age and calendar year, 2001–2011. Age-specific HIV mortality rate ratios for women versus men were estimated in an exponential hazard regression model that included calendar year indicators for each age group and interactions for each age and year with sex. The 45–64 y and 65+ y age groups were combined to improve precision at older ages. The pooled estimate is from a separate regression model. Mortality rate ratios declined after 2004 in all age groups.

Due to differences in age-specific HIV mortality rates for men and women, there is substantial cross-sectional heterogeneity in age-specific female-to-male HIV mortality rate ratios. Women have relatively higher HIV mortality at younger ages; men have higher HIV mortality at older ages. Though we were underpowered to detect age-specific changes, the female-to-male HIV mortality rate ratio appeared to decline in most age groups (Fig 4; S3 Table), and we could not reject the hypothesis of a constant effect across age groups. Female sex has emerged as a protective risk factor for HIV mortality as HIV treatment has become more widely available.

Although men and women had similar rates of HIV mortality at the population level before the ART scale-up, men and women have had starkly different levels of utilization of HIV care and treatment since then. In 2011, 9.0% (95% CI 8.6%, 9.4%) of all women in the population had initiated ART, and an additional 7.9% (95% CI 7.6%, 8.3%) had sought care and had a CD4 count recorded but had not yet initiated ART. Yet just 4.2% (95% CI 3.9%, 4.6%) of all men had initiated ART, and only an additional 2.4% (95% CI 2.1%, 2.6%) of men had sought care but not yet initiated ART (S2 Fig). Over twice as many women had accessed care or initiated ART as men: 16.9% versus 6.6%.

**Fig 5:**
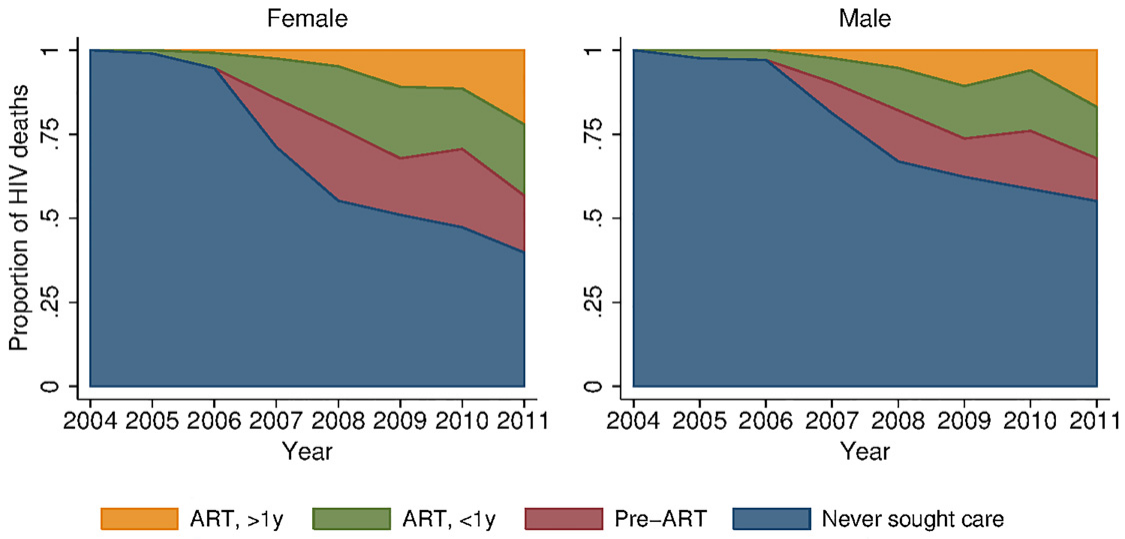
Distribution of HIV deaths across cascade of care, 2001–2011. We excluded all deaths that occurred within 3 mo of migrating into the DSA, as the deceased may not have had the opportunity to seek HIV care in the local health system.

To provide insight into some of the reasons behind these growing female–male disparities, we decomposed deaths due to HIV across different stages in the cascade of care (Figs 5 and S3). In spite of widespread access to ART free of charge at all local clinics, a large proportion of deaths due to HIV occurred among persons who had never sought care for HIV in the public sector treatment program. This phenomenon was particularly pronounced among men. In 2011, 39.8% (95% CI 31.0%, 48.7%) of female HIV-related deaths and 55.1% (95% CI 46.1%, 64.1%) of male HIV-related deaths were among persons who had never sought care (*p*-value for difference in proportions = 0.019). Conditional on seeking care, the distributions of deaths across the other categories—sought care but did not initiate ART (28.2% versus 28.3%), initiated ART less than 1 y ago (35.2% versus 34.0%), initiated ART more than 1 y ago (36.6% versus 37.7%)—were similar for men and women. The proportion of deaths among those who never sought care declined substantially over time for both men and women as access to HIV care and treatment expanded (Fig 5; S4 Table). These results were robust to different definitions of an HIV-related death as diagnosed by verbal autopsy. Restricting the sample to those deaths that were >90% likely to be HIV-related according to the InterVA diagnostic algorithm yielded proportions of 42% and 62% for the percent of HIV deaths occurring in women and men who had never sought care for HIV (S5 Table).

HIV mortality rates were substantially higher for men than for women at each stage of the cascade (Table 3). Among persons who initiated ART at least 1 y prior, HIV mortality was 1.31 per 100 person-years for women and 2.88 per 100 person-years for men in 2011. Rates were 3.37 and 8.49 deaths per 100 person-years for women and men, respectively, in their first year of ART, and 0.76 and 2.45 deaths per 100 person-years for women and men in pre-ART care. Although HIV mortality rates were lowest among persons who had not sought care for HIV—0.21 for women and 0.34 for men—this group represented 83% of women and 93% of men in the population, and hence contributed the largest number of HIV deaths at the population level, as shown in Table 3 and Fig 5.

**Table 3:** HIV mortality across the cascade of care in 2011 (ages 15+).

Parameter	Sex	Care-Seeking Category				Total
		ART >1 y	ART 0–1 y	Pre-ART	Never Sought Care	
Number of HIV-related deaths	Female	26	25	20	47	118
	Male	20	17	15	65	118
Percent of HIV-related deaths (95% CI)	Female	22.0% (14.5, 29.5)	21.2% (13.8, 28.6)	16.9% (10.2, 23.7)	39.8% (31.0, 48.7)	100%
	Male	16.9% (10.2, 23.7)	15.3% (8.7, 21.8)	12.7% (6.7, 18.7)	55.1% (46.1, 64.1)	100%
Percent of at-risk population	Female	6.80%	2.30%	8.10%	82.70%	100%
	Male	3.40%	1.00%	2.30%	93.30%	100%
HIV mortality rate (per 100 person-years)	Female	1.31 (0.89, 1.93)	3.37 (2.07, 5.50)	0.76 (0.47, 1.22)	0.21 (0.16, 0.27)	0.39 (0.33, 0.48)
	Male	2.88 (1.86, 4.47)	8.54 (4.85, 15.04)	2.46 (1.36, 4.44)	0.34 (0.27, 0.44)	0.58 (0.49, 0.70)
Female-to-male HIV mortality rate ratio (age-adjusted)		0.46 (0.25, 0.82)	0.39 (0.19, 0.83)	0.37 (0.17, 0.79)	0.51 (0.35, 0.74)	0.60 (0.46, 0.78)
Female-to-male HIV mortality rate ratio (age- and CD4-adjusted)		0.50 (0.27, 0.92)	0.51 (0.23, 1.12)	0.47 (0.21, 1.03)	—	—

HIV mortality rates were calculated for men and women who were residing in the DSA and at least 15 y on 1 January 2011. Care-seeking category was defined on 1 January 2011; observations were censored on the date when individuals changed care stage. The group “never sought care” includes persons who were not (yet) infected with HIV. Relative rates were computed in an exponential hazard model adjusting for the age categories 15–29, 30–44, 45–64, and 65+ y. Pre-ART CD4-adjusted models were adjusted for log(earliest CD4 count); ART 0–1 y and ART >1 y CD4-adjusted models were adjusted for log(last CD4 count before ART initiation). The female-to-male HIV mortality rate ratio reported here is lower than that reported in Table 2 because sex disparities in HIV mortality were larger for DSA residents compared with non-resident members of the surveillance; non-residents were excluded in the analyses presented in this table.

## Discussion

Mass provision of free ART in public sector facilities in South Africa has coincided with dramatic reductions in HIV-related mortality for both men and women. However, the decline in HIV mortality for women has substantially outpaced the decline for men. Prior to ART scale-up, epidemic HIV had substantially compressed the female–male gap in adult life expectancy because of women’s younger average age at infection and longer lifespan in the absence of HIV. Access to life-prolonging treatment has led not only to the natural decompression of the female–male adult life expectancy gap but also to the unanticipated emergence of male sex as a significant predictive risk factor for HIV mortality at the population level in this setting. Since the start of the ART scale-up in 2004, the female–male gap in adult life expectancy has nearly doubled.

Is the emergent male disadvantage in HIV mortality a fleeting pattern, a blip on the road to universal treatment coverage? Or are we on the path to sustained sex disparities in HIV mortality? To better understand the lingering mortality burden due to HIV, a critical question is where in the HIV cascade of care HIV-related deaths are occurring. By linking data on dates and causes of death from a large population surveillance system with clinical records from South Africa’s national ART program, we decomposed the distribution of HIV deaths in this population across the cascade of care and assessed differences by sex. In spite of the widespread availability of ART, about half of all HIV-related deaths in 2011 occurred among persons who had never sought care in the public sector HIV care and treatment program (55% for men; 40% for women). While previous studies have documented excess mortality among male ART patients [12] and among men receiving pre-ART care [10], this study is the first, to our knowledge, to document the relative significance of HIV mortality among persons who have never sought care and substantial excess mortality among men in this difficult-to-access population.

There are a number of possible explanations for the growing disparity in HIV mortality between men and women. Social norms regarding care-seeking and disclosure of HIV status may present significant barriers to access and adherence for men [31], similar to male care-seeking deficits observed in other settings and for other conditions [32]. Women may be more familiar with local clinics because of their utilization for maternal and child health care. The very successful national program to prevent mother-to-child transmission may encourage HIV-infected women to access and remain in HIV care [33]. In a previous analysis of this cohort, however, accessing care during pregnancy explained only a small fraction of the sex disparity in HIV treatment uptake [8]. Men may also face specific social and cultural barriers to participation in adherence-promoting activities like support groups [34]. Clinics are generally open only during working hours, and with higher employment rates among men than women in this setting [20], this may be a contributing factor. Additionally, high rates of long-distance labor migration may further complicate access to and retention in care for men [35].

We note that higher HIV mortality rates observed for men receiving HIV care and treatment may result from sex differences in adherence and retention behaviors as well as from differences between men and women in the composition of patients seeking care and initiating ART. In previous research on this cohort, we found that men were less likely than women to initiate ART when eligible [8] and that when men did initiate ART, they did so at lower CD4 counts [20]. Delayed care-seeking not only leads to excess mortality among men who have never sought care, but also likely contributes to the excess mortality among men observed at each subsequent stage in the cascade of care through its impact on the composition of patients at each stage. CD4 count at entry into each stage of the cascade offers a noisy, but useful, proxy for mortality risk. Although the male disadvantage in HIV mortality was somewhat attenuated after controlling for CD4 count, men were still twice as likely to die from HIV at each stage in the cascade.

We have documented that a substantial proportion of HIV-related deaths occur among people who have never sought care. Further research is needed to understand the reasons that people dying from HIV choose not to seek lifesaving treatment that is widely available and free of charge. Given the higher risk faced by men, population-wide interventions could be implemented to educate men about the benefits of timely ART initiation for one’s own health [36] and economic productivity [20,37,38], the economic well-being of the household [39,40], and the health of one’s sex partners [41]. Messages could be targeted to settings that men frequent, e.g., football pitches, workplaces, shabeens, and public taxis. Similarly, interventions could emphasize the normalization of ART through sensitization of men to the large number of men and women currently receiving therapy and the very large number of people (40% of the population) who live with someone who has either sought care for HIV or initiated ART [19]. At the same time, changes in public sector service delivery—e.g., by offering clinic hours on weekends rather than solely during working hours, or delivering antiretroviral drugs to non-clinic pick-up points, such as workplaces or local shops—might reduce the costs of care-seeking and encourage men to seek care[42]. Finally, we note the potential complementarity between interventions at different points in the cascade of care: interventions to reduce mortality and improve quality of life among male patients receiving ART may improve community perceptions of treatment and increase uptake of HIV testing, care, and treatment services [43].

Our study assessed trends in adult life expectancy and HIV-related mortality in an HIV-endemic setting in rural South Africa, in the context of South Africa’s national HIV treatment scale-up. The quality of the data is a major strength of this analysis. Data on dates of death were collected through semi-annual household surveys with >99% response rates [21]. Because the data come from population-based surveillance, we observe individuals regardless of whether they have ever sought clinical care and thus capture HIV mortality in individuals who never linked into care or initiated ART. The population perspective also means that we observe individuals and their survival regardless of whether they are retained in clinical care, a challenge in many clinical cohorts. Individuals even continue to be observed if they migrate out of the surveillance area, so long as they remain members of households under surveillance. Verbal autopsy data were collected by trained nurses for all deaths, with a 93% response rate, and HIV/TB-related deaths were identified using a validated algorithm [22]. Results were robust to different coding definitions of HIV-related deaths, as reported above. Dates of ART initiation and CD4 counts were obtained from complete clinical records for the public sector ART program that serves the surveillance area and were linked using individual identifiers [19].

Our study had some limitations. First, because of the nature of the data, we report on only one rural setting in one province in South Africa. We note, however, that this area is in one of the poorest districts in South Africa and has many features—high unemployment, high migration, complex household arrangements, low private sector utilization for HIV treatment—common to rural areas with high HIV prevalence in southern Africa. Second, we report on trends in adult life expectancy and HIV mortality before and during the scale-up of public sector HIV treatment in South Africa. Although the changes in mortality patterns observed coincided with the beginning of ART scale-up in 2004 and progressive expansion of services through 2011, we cannot rule out that other contemporaneous factors could have influenced population mortality trends, such as survivorship effects. In a previous analysis, we simulated the counterfactual path of the epidemic and demonstrated that no rapid increase in life expectancy was predicted based on the internal dynamics of the HIV epidemic in the absence of ART scale-up [1]. Additionally, HIV-cause-deleted adult life expectancy, a summary measure of non-HIV-related mortality, was relatively constant for both men and women across the study period. Third, our data on the cascade of HIV treatment services exclude patients seeking care in the private sector. Though a limitation, we note that private sector care-seeking for HIV treatment is rare in this setting, given the free provision of ART in the public sector. Fourth, we divided the treatment cascade into four discrete, easily defined stages: never had a CD4 count recorded, had a CD4 count recorded but never initiated ART, initiated ART less than a year ago, and initiated ART more than a year ago. These definitions may be conservative: persons who sought care or initiated ART may not still be in care. Investigating sex-specific patterns of clinical attrition and churning, and their contribution to survival disparities between men and women on ART, is an important topic for future research.

As with all verbal autopsy approaches, our method of identifying HIV-related deaths may have resulted in some misclassification. The quality of verbal autopsy data depends on the skill of the interviewer, the willingness of household members to participate, the accuracy of their recall, and the validity of the algorithm used to make diagnoses based on reported symptoms. The underlying data for verbal autopsy at Africa Centre come from high-frequency (twice- and later thrice-annual) demographic surveillance, with autopsy interviews conducted by trained nurses on average 6 mo following a recorded death; in contrast, other surveys have used lay health workers or field interview staff to conduct verbal autopsies and/or relied on longer recall intervals following a death [24,25]. Response rates were very high, with 93% of all deaths assigned a cause of death by verbal autopsy. An active literature has sought to develop and validate verbal autopsy classification algorithms [22,25,44,45]. We used an algorithm (InterVA) that is widely used and has been shown to have high validity in identifying HIV/TB-related deaths in populations with high HIV prevalence [22,24,25]. In particular, the algorithm has been validated locally against physician-coded verbal autopsies in the Africa Centre’s demographic surveillance [22]. TB is a common opportunistic infection in HIV-infected persons, and it can be difficult to distinguish a TB-related death from an HIV-related death. Following previous studies [1,22,25], we coded all TB-related deaths as HIV-related. We note as a limitation that the inclusion of TB-related deaths may somewhat overstate the total number of HIV-related deaths, but this effect is likely to be small: a prior analysis of the Africa Centre surveillance calculated that out of the 53.5% of all adult deaths due to HIV or TB, just 5.3% were attributable to TB without HIV [46]. Further, the inclusion of TB-related mortality is appropriate given the potential spillover effect that ART scale-up may have in reducing the population burden of active TB and transmission of TB to HIV-uninfected persons [47]. Our results were robust to a variety of alternate codings of HIV-related deaths based on the InterVA algorithm.

As a final limitation, we note that our estimates of HIV-cause-deleted life expectancy are interpretable as counterfactual life expectancy in a world without HIV mortality only if HIV and non-HIV causes of death are indeed independent. Although we cannot test this formally (individuals can die only once), we find supporting evidence from several sources. The near-normal life expectancy of patients on ART in previous studies suggests that HIV patients do not face substantially higher or lower mortality rates due to other causes than the general population. The stability of our observed trends in HIV-cause-deleted life expectancy during a period of rapidly falling HIV mortality is also consistent with such an interpretation. Finally, in sensitivity analyses, we found that even very large violations of the independence assumption would not substantially affect our inferences about HIV-cause-deleted life expectancy. Though we cannot rule out dependence across causes, our estimates of HIV-cause-deleted life expectancy likely provide a reasonable estimate of the potential adult life expectancy that would be observed with the elimination of HIV mortality, e.g., through further advances in treatment and prevention (see S1 Text for further discussion of these points).

This paper offers a population perspective on the gendered impacts of mass HIV treatment [12,16,48,49]. Though population adult life expectancy has increased dramatically for women during the scale-up of ART, the gains for men have been more modest. This difference is partially explained by the fact that women had a larger number of years of life lost due to HIV than men prior to treatment scale-up; ART has led to a “natural” decompression in the female–male life expectancy gap as HIV mortality has declined. Yet this is not the whole story. HIV mortality has declined significantly faster among women than among men, and male sex has emerged as a risk factor for HIV mortality in this setting. A high proportion of HIV-related deaths occurred among men who had never sought care or treatment for HIV in the public sector, despite the fact that treatment is widely available and free at point of service. Gender-targeted programming has often sought to improve health for women and girls. Our results suggest that further research to understand male deficits in care-seeking and to design effective interventions to increase uptake of HIV services among men is needed to realize the full benefits of mass ART provision. Without better outreach to men, achievement of the Joint United Nations Programme on HIV/AIDS 2020 targets of 90% tested, 90% initiated, and 90% virally suppressed [50] is unlikely. Additionally, given the prevention benefits of ART, male-sensitive programming may be necessary to reduce the burden of HIV disease among men and women alike.

## Supporting Information

S1 Fig Declines in adult life expectancy due to HIV are associated with declines in the female–male adult life expectancy gap, 1990–2000. Countries with >10% adult HIV prevalence are highlighted in red. Countries with high HIV prevalence suffered large losses in adult life expectancy during the 1990s, when HIV infection spread rapidly. In many—but not all—of these countries, adult life expectancy declined more among women than among men. Liberia and Eritrea were extreme outliers and were excluded to preserve the scale of the figure; these two countries had very large increases in male adult life expectancy coinciding with the end of violent conflicts. Source: authors’ analysis of published WHO data [5].
(EPS)

S2 Fig Proportion of male and female DSA residents ages 15 y and over who sought care for HIV and/or initiated ART in the public sector treatment program. Proportion of DSA residents ages 15 and over who ever sought care for HIV (left) or initiated ART (right). In 2011, 9% of all women had initiated ART, and an additional 8% had sought care and had a recorded CD4 count but had not yet initiated ART. Just 4% of all men had initiated ART; an additional 3% had sought care but not yet initiated ART.
(EPS)

S3 Fig Distribution of HIV deaths across the cascade of care, 2011. We excluded all deaths that occurred less than 3 mo after migration into to the DSA.
(EPS)

S1 Table Risk table for continuous-time Kaplan-Meier survival curves (Fig 3). (DOCX)

S2 Table Relative rate of HIV death, 2001–2011: regression results. (DOCX)

S3 Table HIV mortality rates by age, sex, and year, 2001–2011. (DOCX)

S4 Table HIV deaths by year and type for individuals ages 15 y and over. (DOCX)

S5 Table Proportion of HIV-related deaths in 2011 among individuals who never sought care, using alternate definitions of HIV-related deaths. (DOCX)

S1 Text Sensitivity analysis. (DOCX)

S2 Text Adherence to analysis protocol. (DOCX)

S3 Text STROBE checklist. (DOC)

## Abbreviations

Africa Centre: Africa Centre for Population Health
ART: antiretroviral therapy
DSA: demographic surveillance area
RR: rate ratio
TB: tuberculosis

## References

[1] Bor J, Herbst A, Newell M, Bärnighausen T. Increases in adult life expectancy in rural South Africa: valuing the scale-up of HIV treatment. Science. 2013;339:961–965. 10.1126/science.1230413 23430655 PMC3860268

[2] Reniers G, Eaton J, Nakiyingi-Miiro J, Crampin A, Kabudula C, Herbst K, et al. The impact of antiretroviral therapy on adult life expectancy in sub-Saharan Africa [abstract]. Conference on Retroviruses and Opportunistic Infections (CROI 2015); 23–26 Feb 2015; Seattle, WA, US.

[3] Lozano R, Naghavi M, Foreman K, Lim S, Shibuya K, Aboyans V, et al Global and regional mortality from 235 causes of death for 20 age groups in 1990 and 2010: a systematic analysis for the Global Burden of Disease Study 2010. Lancet. 2012;380:2095–2128. 10.1016/S0140-6736(12)61728-0 23245604 PMC10790329

[4] Pillay Y. Operational and programmatic considerations in scaling up ART [abstract]. 7th International AIDS Society Conference on HIV Pathogenesis, Treatment and Prevention; 30 Jun–3 Jul 2013; Kuala Lumpur, Malaysia.

[5] World Health Organization. World health statistics 2012 Geneva: World Health Organization; 2012.

[6] Shisana O, Rehle T, Simbayi L, Zuma K, Jooste S, Zungu N, et al South African national HIV prevalence, incidence and behaviour survey, 2012 Cape Town: Human Sciences Research Council; 2014. 10.2989/16085906.2016.1153491 27002359

[7] Mugglin C, Estill J, Wandeler G, Bender N, Egger M, Gsponer T, et al Loss to programme between HIV diagnosis and initiation of antiretroviral therapy in sub-Saharan Africa: systematic review and meta-analysis. Trop Med Int Health. 2012;17:1509–1520. 10.1111/j.1365-3156.2012.03089.x 22994151 PMC3895621

[8] Bärnighausen T, Tanser F, Herbst K, Mutevedzi T, Mossong J, Newell M. Structural barriers to antiretroviral treatment: a study using population-based CD4 cell count and linked antiretroviral treatment programme data [abstract]. Lancet. 2013;382(S5):3935 10.1016/S0140-6736(13)62253-9

[9] Bassett I, Regan S, Luthuli P, Mbonambi H, Bearnot B, Pendleton A, et al Linkage to care following community-based mobile HIV testing compared with clinic-based testing in Umlazi Township, Durban, South Africa. HIV Med. 2014;15:367–372. 10.1111/hiv.12115 24251725 PMC4026348

[10] Lessells RJ, Mutevedzi PC, Cooke GS, Newell ML. Retention in HIV care for individuals not yet eligible for antiretroviral therapy: rural KwaZulu-Natal, South Africa. J Acquir Immune Defic Syndr. 2011;56:e79 10.1097/QAI.0b013e3182075ae2 21157360 PMC3073481

[11] Hawkins C, Chalamilla G, Okuma J, Spiegelman D, Hertzmark E, Aris E, et al Sex differences in antiretroviral treatment outcomes among HIV-infected adults in an urban Tanzanian setting. AIDS. 2011;25:1189–1197. 10.1097/QAD.0b013e3283471deb 21505309

[12] Cornell M, Schomaker M, Garone DB, Giddy J, Hoffmann CJ, Lessells R, et al Gender differences in survival among adult patients starting antiretroviral therapy in South Africa: a multicentre cohort study. PLoS Med. 2012;9:e1001304 10.1371/journal.pmed.1001304 22973181 PMC3433409

[13] Lahuerta M, Wu Y, Hoffman S, Elul B, Kulkarni SG, Remien RH, et al Advanced HIV disease at entry into HIV care and initiation of antiretroviral therapy during 2006–2011: findings from four sub-saharan African countries. Clin Infect Dis. 2014;58:432–441. 10.1093/cid/cit724 24198226 PMC3890338

[14] Kranzer K, Lewis JJ, Ford N, Zeinecker J, Orrell C, Lawn SD, et al Treatment interruption in a primary care antiretroviral therapy programme in South Africa: cohort analysis of trends and risk factors. J Acquir Immune Defic Syndr. 2010;55:e17–e23. 10.1097/QAI.0b013e3181f275fd 20827216 PMC3024539

[15] Mills EJ, Bakanda C, Birungi J, Chan K, Hogg RS, Ford N, et al Male gender predicts mortality in a large cohort of patients receiving antiretroviral therapy in Uganda. J Int AIDS Soc. 2011;14:52 10.1186/1758-2652-14-52 22050673 PMC3220631

[16] Druyts E, Dybul M, Kanters S, Nachega J, Birungi J, Ford N, et al Male sex and the risk of mortality among individuals enrolled in antiretroviral therapy programs in Africa: a systematic review and meta-analysis. AIDS. 2013;27:417–425. 10.1097/QAD.0b013e328359b89b 22948271

[17] Maskew M, Brennan AT, Westreich D, McNamara L, MacPhail AP, Fox MP. Gender differences in mortality and CD4 count response among virally suppressed HIV-positive patients. J Womens Health (Larchmt). 2013;22:113–120. 10.1089/jwh.2012.3585 23350862 PMC3579326

[18] Zaidi J, Grapsa E, Tanser F, Newell M-L, Bärnighausen T. Dramatic increase in HIV prevalence after scale-up of antiretroviral treatment. AIDS. 2013;27:2301–2305. 10.1097/QAD.0b013e328362e832 23669155 PMC4264533

[19] Bor J, Bärnighausen T, Newell C, Tanser F, Newell ML. Social exposure to an antiretroviral treatment programme in rural KwaZulu‐Natal. Trop Med Int Health. 2011;16:988–994. 10.1111/j.1365-3156.2011.02795.x 21615631 PMC4295018

[20] Bor J, Tanser F, Newell M-L, Bärnighausen T. In a study of a population cohort in South Africa, HIV patients on antiretrovirals had nearly full recovery of employment. Health Aff (Millwood). 2012;31:1459–1469. 10.1377/hlthaff.2012.0407 22778335 PMC3819460

[21] Tanser F, Hosegood V, Bärnighausen T, Herbst K, Nyirenda M, Muhwava W, et al Cohort profile: Africa Centre Demographic Information System (ACDIS) and population-based HIV survey. Int J Epidemiol. 2008;37:956–962. 10.1093/ije/dym211 17998242 PMC2557060

[22] Herbst AJ, Mafojane T, Newell M-L. Verbal autopsy-based cause-specific mortality trends in rural KwaZulu-Natal, South Africa, 2000–2009. Popul Health Metr. 2011;9:47 10.1186/1478-7954-9-47 21819602 PMC3160940

[23] Herbst AJ, Cooke GS, Bärnighausen T, KanyKany A, Tanser F, Newell M-L. Adult mortality and antiretroviral treatment roll-out in rural KwaZulu-Natal, South Africa. Bull World Health Organ. 2009;87:754–762. 19876542 10.2471/BLT.08.058982 PMC2755311

[24] Byass P, Calvert C, Miiro-Nakiyingi J, Lutalo T, Michael D, Crampin A, et al InterVA-4 as a public health tool for measuring HIV/AIDS mortality: a validation study from five African countries. Glob Health Action. 2013;6:22448 10.3402/gha.v6i0.22448 24138838 PMC3800746

[25] Byass P, Herbst K, Fottrell E, Ali MM, Odhiambo F, Amek N, et al Comparing verbal autopsy cause of death findings as determined by physician coding and probabilistic modelling: a public health analysis of 54 000 deaths in Africa and Asia. J Glob Health. 2015;5:010402 10.7189/jogh.05.010402 25734004 PMC4337147

[26] Bärnighausen T, Bor J, Wandira-Kazibwe S, Canning D. Correcting HIV prevalence estimates for survey nonparticipation using Heckman-type selection models. Epidemiology. 2011;22:27–35. 10.1097/EDE.0b013e3181ffa201 21150352

[27] Mills EJ, Bakanda C, Birungi J, Chan K, Ford N, Cooper CL, et al Life expectancy of persons receiving combination antiretroviral therapy in low-income countries: a cohort analysis from Uganda. Ann Intern Med. 2011;155:209–217. 10.7326/0003-4819-155-4-201108160-00358 21768555

[28] Johnson LF, Mossong J, Dorrington RE, Schomaker M, Hoffmann CJ, Keiser O, et al Life expectancies of South African adults starting antiretroviral treatment: collaborative analysis of cohort studies. PLoS Med. 2013;10:e1001418 10.1371/journal.pmed.1001418 23585736 PMC3621664

[29] Welaga P, Hosegood V, Weiner R, Hill C, Herbst K, Newell M-L. Coming home to die? the association between migration and mortality in rural South Africa. BMC Public Health. 2009;9:193 10.1186/1471-2458-9-193 19538717 PMC2706824

[30] Garrib A, Herbst AJ, Hosegood V, Newell M-L. Injury mortality in rural South Africa 2000–2007: rates and associated factors. Trop Med Int Health. 2011;16:439–446. 10.1111/j.1365-3156.2011.02730.x 21284789 PMC3085120

[31] Schneider H, Govender V, Harris B, Cleary S, Moshabela M, Birch S. Gender differences in experiences of ART services in South Africa: a mixed methods study. Trop Med Int Health. 2012;17:820–826. 10.1111/j.1365-3156.2012.03009.x 22594691

[32] Galdas PM, Cheater F, Marshall P. Men and health help-seeking behaviour: literature review. J Adv Nurs. 2005;49:616–623. 10.1111/j.1365-2648.2004.03331.x 15737222

[33] Goga AE, Dinh T-H, Jackson DJ, Lombard C, Delaney KP, Puren A, et al First population-level effectiveness evaluation of a national programme to prevent HIV transmission from mother to child, South Africa. J Epidemiol Community Health. 2014;69:240–248. 10.1136/jech-2014-204535 25371480 PMC4345523

[34] Madiba S, Canti-Sigaqa V. Barriers to participate in support groups for people living with HIV: A qualitative study with men receiving antiretroviral treatment in a HIV clinic in Mthatha, South Africa. Glob J Health Sci. 2012;4:119–128. 10.5539/gjhs.v4n6p119 23121748 PMC4776954

[35] Camlin CS, Hosegood V, Newell M-L, McGrath N, Bärnighausen T, Snow RC. Gender, migration and HIV in rural KwaZulu-Natal, South Africa. PLoS ONE. 2010;5:e11539 10.1371/journal.pone.0011539 20634965 PMC2902532

[36] Bor J, Moscoe E, Mutevedzi P, Newell M-L, Bärnighausen T. Regression discontinuity designs in epidemiology: causal inference without randomized trials. Epidemiology. 2014;25:729–737. 10.1097/EDE.0000000000000138 25061922 PMC4162343

[37] Thirumurthy H, Zivin JG, Goldstein M. The economic impact of AIDS treatment: labor supply in western Kenya. J Hum Resour. 2008;43:511–552. 22180664 PMC3237059

[38] Larson BA, Fox MP, Rosen S, Bii M, Sigei C, Shaffer D, et al Do the socioeconomic impacts of antiretroviral therapy vary by gender? A longitudinal study of Kenyan agricultural worker employment outcomes. BMC Public Health. 2009;9:240 10.1186/1471-2458-9-240 19604381 PMC2717954

[39] Zivin JG, Thirumurthy H, Goldstein M. AIDS treatment and intrahousehold resource allocation: children’s nutrition and schooling in Kenya. J Public Econ. 2009;93:1008–1015. 22180689 10.1016/j.jpubeco.2009.03.003 PMC3238680

[40] Bor J, Tanser F, Newell M-L, Bärnighausen T. Economic spillover effects of ART on rural South African households [abstract]. International AIDS and Economics Network 7th AIDS and Economics Pre-Conference; 20–21 Jul 2012; Washington, DC, US.

[41] Cohen MS, Chen YQ, McCauley M, Gamble T, Hosseinipour MC, Kumarasamy N, et al Prevention of HIV-1 infection with early antiretroviral therapy. N Engl J Med. 2011;365:493–505. 10.1056/NEJMoa1105243 21767103 PMC3200068

[42] Chimbindi N, Bor J, Newell ML, Tanser F, Baltussen R, Hontelez J, et al Time and money: the true costs of health care utilization for patients receiving “free” HIV/tuberculosis care and treatment in rural KwaZulu-Natal. JAIDS. 2015;70(2):e52–e60. 26371611 10.1097/QAI.0000000000000728 PMC4748708

[43] Wilson N. Antiretroviral therapy and demand for HIV testing: evidence from Zambia. 1 8 2011 Social Science Research Network. 10.2139/ssrn.1982185 26970992

[44] Lozano R, Freeman MK, James SL, Campbell B, Lopez AD, Flaxman AD, et al Performance of InterVA for assigning causes of death to verbal autopsies: multisite validation study using clinical diagnostic gold standards. Popul Health Metr. 2011;9:50 10.1186/1478-7954-9-50 21819580 PMC3160943

[45] Murray CJL, Lozano R, Flaxman AD, Serina P, Phillips D, Stewart A, et al Using verbal autopsy to measure causes of death: the comparative performance of existing methods. BMC Med. 2014;12:5 10.1186/1741-7015-12-5 24405531 PMC3891983

[46] Hosegood V, Vanneste A-M, Timaeus IM. Levels and causes of adult mortality in rural South Africa: the impact of AIDS. AIDS. 2004;18:663–671. 10.1097/01.aids.0000111463.61782.aa 15090772

[47] Lawn SD, Harries AD, Williams BG, Chaisson RE, Losina E, De Cock KM, et al Antiretroviral therapy and the control of HIV-associated tuberculosis. Will ART do it? Int J Tuberc Lung Dis. 2011;15:571–581. 10.5588/ijtld.10.0483 21756508 PMC4067901

[48] Cornell M, McIntyre J, Myer L. Men and antiretroviral therapy in Africa: our blind spot. Trop Med Int Health. 2011;16:828–829. 10.1111/j.1365-3156.2011.02767.x 21418449 PMC3749374

[49] Cornell M, Myer L. Men and mortality: sex inequality in health outcomes. AIDS. 2013;27:849–850. 10.1097/QAD.0b013e32835e399b 23719356

[50] Joint United Nations Programme on HIV/AIDS. 90-90-90: an ambitious treatment target to help end the AIDS epidemic Geneva: Joint United Nations Programme on HIV/AIDS; 2014 Available: http://www.unaids.org/sites/default/files/media_asset/90-90-90_en_0.pdf. Accessed 20 October 2015.

