# Supplementary material for: Mass HIV Treatment and Sex Disparities in Life Expectancy: Demographic Surveillance in Rural South Africa
Source: PLoS Med. 2015 Nov 24;12(11):e1001905. doi: 10.1371/journal.pmed.1001905 (PMC4658174; doi:10.1371/journal.pmed.1001905)
Supplement: S1 Table — (DOCX) [file pmed.1001905.s004.docx]

**S1 Table. Risk table for Kaplan-Meier period survival curves (Fig 3).**

| **WOMEN** | **Adult LE, 2003** | | | **Adult LE, 2011** | | | **HIV-cause-deleted adult LE, 2001-2011** | | |
| --- | --- | --- | --- | --- | --- | --- | --- | --- | --- |
| ***Age*** | ***At Risk*** | ***Died*** | ***S(t+1)*** | ***At Risk*** | ***Died*** | ***S(t+1)*** | ***At Risk*** | ***Died*** | ***S(t+1)*** |
| **15** | 1082 | 3 | 100% | 1079 | 3 | 100% | 12442 | 9 | 100% |
| **16** | 1097 | 1 | 100% | 1095 | 1 | 100% | 12532 | 15 | 100% |
| **17** | 1200 | 2 | 99% | 1190 | 4 | 99% | 12487 | 7 | 100% |
| **18** | 1125 | 5 | 99% | 1166 | 3 | 99% | 12361 | 14 | 100% |
| **19** | 1097 | 7 | 98% | 1091 | 3 | 99% | 12037 | 16 | 100% |
| **20** | 1135 | 8 | 98% | 1078 | 6 | 98% | 11807 | 18 | 99% |
| **21** | 902 | 8 | 97% | 1143 | 5 | 98% | 11496 | 18 | 99% |
| **22** | 897 | 10 | 96% | 1039 | 7 | 97% | 11050 | 28 | 99% |
| **23** | 907 | 18 | 94% | 1033 | 2 | 97% | 10649 | 23 | 99% |
| **24** | 771 | 13 | 93% | 1001 | 9 | 96% | 10280 | 29 | 98% |
| **25** | 790 | 17 | 91% | 1082 | 6 | 96% | 9918 | 28 | 98% |
| **26** | 832 | 17 | 89% | 974 | 11 | 95% | 9445 | 33 | 98% |
| **27** | 780 | 14 | 87% | 973 | 6 | 94% | 8884 | 30 | 98% |
| **28** | 789 | 25 | 84% | 942 | 8 | 93% | 8451 | 31 | 97% |
| **29** | 627 | 23 | 82% | 771 | 10 | 92% | 7844 | 28 | 97% |
| **30** | 692 | 22 | 79% | 735 | 6 | 91% | 7474 | 35 | 96% |
| **31** | 519 | 18 | 77% | 752 | 14 | 90% | 7119 | 29 | 96% |
| **32** | 624 | 25 | 73% | 583 | 15 | 88% | 6768 | 38 | 96% |
| **33** | 588 | 22 | 71% | 571 | 8 | 87% | 6430 | 23 | 95% |
| **34** | 600 | 20 | 69% | 600 | 4 | 86% | 6103 | 27 | 95% |
| **35** | 424 | 20 | 66% | 584 | 6 | 85% | 5842 | 21 | 94% |
| **36** | 496 | 13 | 64% | 569 | 7 | 84% | 5602 | 20 | 94% |
| **37** | 504 | 20 | 61% | 502 | 5 | 83% | 5325 | 27 | 94% |
| **38** | 473 | 12 | 60% | 537 | 11 | 82% | 5182 | 13 | 93% |
| **39** | 466 | 3 | 59% | 362 | 6 | 81% | 4920 | 21 | 93% |
| **40** | 490 | 14 | 58% | 443 | 6 | 79% | 4881 | 14 | 93% |
| **41** | 390 | 14 | 56% | 450 | 5 | 78% | 4732 | 18 | 92% |
| **42** | 447 | 16 | 54% | 434 | 4 | 78% | 4649 | 20 | 92% |
| **43** | 456 | 13 | 52% | 298 | 7 | 76% | 4521 | 22 | 92% |
| **44** | 471 | 12 | 51% | 358 | 2 | 76% | 4410 | 13 | 91% |
| **45** | 380 | 8 | 50% | 382 | 4 | 75% | 4221 | 22 | 91% |
| **46** | 307 | 12 | 48% | 380 | 5 | 74% | 4045 | 24 | 90% |
| **47** | 270 | 5 | 47% | 377 | 2 | 74% | 3836 | 17 | 90% |
| **48** | 311 | 7 | 46% | 393 | 8 | 72% | 3652 | 20 | 89% |
| **49** | 236 | 9 | 45% | 312 | 4 | 71% | 3339 | 19 | 89% |
| **50** | 287 | 5 | 44% | 356 | 6 | 70% | 3246 | 32 | 88% |
| **51** | 162 | 6 | 43% | 356 | 5 | 69% | 3133 | 25 | 87% |
| **52** | 283 | 6 | 41% | 370 | 5 | 68% | 2964 | 26 | 87% |
| **53** | 285 | 7 | 41% | 299 | 3 | 68% | 2719 | 27 | 86% |
| **54** | 262 | 5 | 40% | 250 | 8 | 66% | 2529 | 23 | 85% |
| **55** | 179 | 5 | 39% | 218 | 1 | 65% | 2406 | 15 | 85% |
| **56** | 178 | 5 | 38% | 258 | 3 | 65% | 2256 | 30 | 84% |
| **57** | 164 | 3 | 37% | 192 | 3 | 64% | 2128 | 23 | 83% |
| **58** | 113 | 6 | 36% | 226 | 6 | 62% | 2045 | 37 | 81% |
| **59** | 166 | 3 | 35% | 131 | 4 | 61% | 1975 | 17 | 81% |
| **60** | 178 | 9 | 33% | 230 | 8 | 58% | 2029 | 42 | 79% |
| **61** | 188 | 4 | 32% | 222 | 3 | 57% | 1991 | 27 | 78% |
| **62** | 242 | 5 | 32% | 205 | 2 | 57% | 1887 | 37 | 76% |
| **63** | 221 | 10 | 30% | 139 | 3 | 56% | 1778 | 37 | 75% |
| **64** | 161 | 6 | 29% | 140 | 4 | 54% | 1749 | 37 | 73% |
| **65** | 136 | 8 | 28% | 121 | 3 | 53% | 1660 | 42 | 71% |
| **66** | 139 | 5 | 27% | 88 | 3 | 51% | 1661 | 40 | 70% |
| **67** | 109 | 5 | 26% | 126 | 3 | 50% | 1679 | 39 | 68% |
| **68** | 171 | 3 | 25% | 130 | 2 | 49% | 1697 | 37 | 67% |
| **69** | 150 | 3 | 25% | 143 | 4 | 47% | 1626 | 45 | 65% |
| **70** | 159 | 7 | 24% | 186 | 4 | 46% | 1678 | 45 | 63% |
| **71** | 116 | 3 | 23% | 171 | 10 | 44% | 1582 | 44 | 62% |
| **72** | 246 | 9 | 22% | 115 | 9 | 41% | 1472 | 64 | 59% |
| **73** | 134 | 11 | 21% | 101 | 2 | 41% | 1407 | 57 | 57% |
| **74** | 97 | 8 | 20% | 103 | 3 | 39% | 1335 | 65 | 54% |
| **75** | 103 | 9 | 18% | 76 | 3 | 38% | 1239 | 60 | 52% |
| **76** | 94 | 4 | 17% | 116 | 5 | 36% | 1152 | 63 | 49% |
| **77** | 69 | 4 | 16% | 93 | 3 | 35% | 1019 | 40 | 47% |
| **78** | 56 | 5 | 15% | 109 | 7 | 33% | 903 | 54 | 45% |
| **79** | 33 | 3 | 14% | 66 | 7 | 31% | 764 | 57 | 42% |
| **80** | 39 | 1 | 14% | 144 | 6 | 29% | 707 | 42 | 39% |
| **81** | 27 | 3 | 12% | 73 | 11 | 26% | 567 | 35 | 37% |
| **82** | 56 | 4 | 11% | 60 | 2 | 25% | 528 | 32 | 35% |
| **83** | 28 | 6 | 10% | 60 | 7 | 23% | 447 | 41 | 32% |
| **84** | 57 | 2 | 9% | 54 | 6 | 20% | 379 | 30 | 30% |
| **85** | 12 | 7 | 7% | 46 | 4 | 19% | 300 | 30 | 27% |
| **86** | 18 | 1 | 7% | 24 | 1 | 18% | 265 | 23 | 25% |
| **87** | 5 | 1 | 6% | 17 | 3 | 16% | 225 | 30 | 22% |
| **88** | 28 | 3 | 5% | 21 | 3 | 14% | 194 | 26 | 20% |
| **89** | 16 | 4 | 4% | 12 | 0 | 14% | 172 | 12 | 18% |
| **90** | 14 | 4 | 3% | 14 | 2 | 11% | 168 | 25 | 16% |
| **91** | 8 | 3 | 2% | 14 | 0 | 11% | 158 | 19 | 14% |
| **92** | 24 | 2 | 2% | 25 | 0 | 11% | 133 | 17 | 13% |
| **93** | 14 | 3 | 2% | 5 | 1 | 11% | 96 | 18 | 11% |
| **94** | 5 | 3 | 1% | 7 | 1 | 8% | 82 | 17 | 9% |

| **MEN** | **Adult LE, 2003** | | | **Adult LE, 2011** | | | **HIV-cause-deleted adult LE, 2001-2011** | | |
| --- | --- | --- | --- | --- | --- | --- | --- | --- | --- |
| ***Age*** | ***At Risk*** | ***Died*** | ***S(t+1)*** | ***At Risk*** | ***Died*** | ***S(t+1)*** | ***At Risk*** | ***Died*** | ***S(t+1)*** |
| **15** | 1115 | 0 | 100% | 1120 | 2 | 100% | 12372 | 7 | 100% |
| **16** | 1120 | 0 | 100% | 1141 | 1 | 100% | 12310 | 13 | 100% |
| **17** | 1134 | 2 | 100% | 1082 | 1 | 100% | 12199 | 22 | 100% |
| **18** | 1127 | 4 | 99% | 1098 | 2 | 99% | 12179 | 29 | 99% |
| **19** | 1062 | 6 | 99% | 1042 | 2 | 99% | 11823 | 36 | 99% |
| **20** | 1075 | 4 | 99% | 1163 | 2 | 99% | 11535 | 37 | 99% |
| **21** | 825 | 8 | 98% | 1077 | 1 | 99% | 11182 | 36 | 98% |
| **22** | 855 | 8 | 97% | 1029 | 6 | 98% | 10644 | 36 | 98% |
| **23** | 867 | 4 | 96% | 1019 | 5 | 98% | 10261 | 50 | 98% |
| **24** | 699 | 10 | 95% | 1029 | 2 | 98% | 9829 | 47 | 97% |
| **25** | 785 | 12 | 94% | 1014 | 9 | 97% | 9371 | 55 | 97% |
| **26** | 690 | 8 | 93% | 994 | 10 | 96% | 8793 | 55 | 96% |
| **27** | 703 | 12 | 91% | 928 | 10 | 95% | 8296 | 42 | 96% |
| **28** | 676 | 25 | 88% | 887 | 13 | 94% | 7856 | 53 | 95% |
| **29** | 602 | 20 | 85% | 707 | 9 | 93% | 7189 | 54 | 94% |
| **30** | 660 | 14 | 83% | 709 | 12 | 91% | 6777 | 49 | 94% |
| **31** | 466 | 27 | 79% | 692 | 13 | 89% | 6379 | 51 | 93% |
| **32** | 475 | 23 | 75% | 550 | 10 | 88% | 6011 | 49 | 92% |
| **33** | 515 | 22 | 72% | 598 | 13 | 86% | 5693 | 51 | 91% |
| **34** | 491 | 21 | 69% | 532 | 14 | 84% | 5321 | 35 | 91% |
| **35** | 381 | 26 | 65% | 539 | 7 | 83% | 5004 | 36 | 90% |
| **36** | 364 | 19 | 62% | 495 | 11 | 81% | 4705 | 50 | 89% |
| **37** | 418 | 13 | 60% | 433 | 11 | 79% | 4495 | 42 | 88% |
| **38** | 382 | 19 | 57% | 458 | 11 | 77% | 4341 | 32 | 88% |
| **39** | 437 | 17 | 55% | 316 | 4 | 76% | 4059 | 36 | 87% |
| **40** | 417 | 21 | 52% | 335 | 10 | 74% | 3917 | 36 | 86% |
| **41** | 301 | 18 | 50% | 368 | 6 | 73% | 3765 | 31 | 86% |
| **42** | 357 | 11 | 48% | 348 | 8 | 71% | 3592 | 37 | 85% |
| **43** | 318 | 13 | 46% | 245 | 3 | 70% | 3443 | 33 | 84% |
| **44** | 302 | 14 | 44% | 267 | 13 | 67% | 3327 | 38 | 83% |
| **45** | 321 | 15 | 42% | 293 | 6 | 66% | 3218 | 33 | 82% |
| **46** | 257 | 11 | 41% | 244 | 9 | 64% | 3090 | 29 | 81% |
| **47** | 266 | 16 | 38% | 296 | 9 | 61% | 2951 | 40 | 80% |
| **48** | 284 | 8 | 37% | 281 | 10 | 59% | 2830 | 29 | 80% |
| **49** | 209 | 12 | 35% | 221 | 9 | 57% | 2590 | 44 | 78% |
| **50** | 257 | 8 | 34% | 242 | 0 | 57% | 2510 | 36 | 77% |
| **51** | 164 | 9 | 33% | 207 | 7 | 56% | 2374 | 42 | 76% |
| **52** | 202 | 8 | 31% | 202 | 6 | 54% | 2232 | 44 | 74% |
| **53** | 227 | 7 | 30% | 211 | 5 | 53% | 2096 | 41 | 73% |
| **54** | 154 | 11 | 29% | 176 | 6 | 51% | 1927 | 38 | 72% |
| **55** | 142 | 4 | 28% | 171 | 7 | 49% | 1803 | 28 | 71% |
| **56** | 129 | 6 | 27% | 185 | 4 | 48% | 1687 | 32 | 69% |
| **57** | 123 | 7 | 25% | 157 | 2 | 47% | 1573 | 34 | 68% |
| **58** | 109 | 5 | 24% | 158 | 6 | 46% | 1481 | 33 | 66% |
| **59** | 122 | 3 | 24% | 128 | 3 | 45% | 1416 | 32 | 65% |
| **60** | 139 | 5 | 23% | 136 | 5 | 43% | 1367 | 41 | 63% |
| **61** | 119 | 4 | 22% | 151 | 7 | 41% | 1283 | 35 | 61% |
| **62** | 138 | 2 | 22% | 108 | 11 | 38% | 1178 | 40 | 60% |
| **63** | 109 | 12 | 20% | 76 | 4 | 36% | 1088 | 44 | 57% |
| **64** | 100 | 4 | 19% | 78 | 4 | 34% | 1044 | 34 | 55% |
| **65** | 89 | 4 | 18% | 86 | 2 | 33% | 981 | 27 | 54% |
| **66** | 67 | 8 | 16% | 67 | 3 | 32% | 908 | 28 | 52% |
| **67** | 60 | 1 | 16% | 64 | 4 | 30% | 846 | 41 | 50% |
| **68** | 58 | 4 | 15% | 93 | 3 | 29% | 800 | 41 | 48% |
| **69** | 54 | 6 | 13% | 75 | 3 | 28% | 688 | 40 | 45% |
| **70** | 60 | 3 | 13% | 86 | 3 | 27% | 665 | 31 | 43% |
| **71** | 25 | 4 | 11% | 60 | 13 | 23% | 615 | 38 | 41% |
| **72** | 91 | 1 | 11% | 55 | 3 | 22% | 585 | 36 | 38% |
| **73** | 67 | 5 | 10% | 38 | 4 | 20% | 542 | 43 | 35% |
| **74** | 55 | 11 | 9% | 25 | 3 | 18% | 499 | 43 | 33% |
| **75** | 58 | 5 | 8% | 34 | 4 | 16% | 494 | 25 | 31% |
| **76** | 39 | 7 | 7% | 26 | 4 | 14% | 455 | 38 | 29% |
| **77** | 42 | 5 | 6% | 26 | 6 | 11% | 409 | 32 | 27% |
| **78** | 34 | 2 | 6% | 34 | 5 | 9% | 366 | 24 | 25% |
| **79** | 17 | 1 | 6% | 14 | 1 | 9% | 322 | 33 | 23% |
| **80** | 13 | 0 | 6% | 49 | 0 | 9% | 318 | 19 | 21% |
| **81** | 19 | 2 | 5% | 25 | 2 | 9% | 267 | 22 | 20% |
| **82** | 29 | 4 | 4% | 27 | 5 | 7% | 226 | 36 | 17% |
| **83** | 16 | 3 | 4% | 18 | 2 | 6% | 185 | 24 | 15% |
| **84** | 20 | 2 | 3% | 12 | 3 | 5% | 156 | 18 | 14% |
| **85** | 5 | 2 | 3% | 22 | 1 | 5% | 133 | 13 | 13% |
| **86** | 2 | 1 | 1% | 17 | 4 | 4% | 112 | 16 | 11% |
| **87** | 2 | 0 | 1% | 7 | 1 | 4% | 89 | 12 | 10% |
| **88** | 11 | 1 | 1% | 4 | 1 | 3% | 69 | 11 | 8% |
| **89** | 4 | 3 | 1% | 6 | 0 | 3% | 62 | 8 | 7% |
| **90** | 1 | 0 | 1% | 9 | 3 | 2% | 54 | 9 | 6% |
| **91** | 2 | 0 | 1% | 8 | 0 | 2% | 46 | 7 | 6% |
| **92** | 3 | 0 | 1% | 8 | 1 | 2% | 29 | 9 | 4% |
| **93** | 4 | 0 | 1% | 3 | 1 | 1% | 18 | 3 | 4% |
| **94** | 1 | 1 | 0% | 0 | 0 | 1% | 18 | 1 | 4% |
