# Supplementary material for: Mass HIV Treatment and Sex Disparities in Life Expectancy: Demographic Surveillance in Rural South Africa
Source: PLoS Med. 2015 Nov 24;12(11):e1001905. doi: 10.1371/journal.pmed.1001905 (PMC4658174; doi:10.1371/journal.pmed.1001905)
Supplement: S2 Table — (DOCX) [file pmed.1001905.s005.docx]

**S1 Table, Relative rate of HIV death, 2001-2011: regression results**

|  | **(1) Crude** | | | **(2) Age-Adjusted** | | | |
| --- | --- | --- | --- | --- | --- | --- | --- |
|  | **RR** | **(95% CI)** | | **RR** | **(95% CI)** | | |
| 2001 | 1.00 | 0.82 | 1.21 | 1.01 | 0.83 | | 1.22 |
| 2002 | 0.93 | 0.77 | 1.13 | 0.94 | 0.77 | | 1.13 |
| 2003 | 1.00 | Ref group | | 1.00 | Ref group | | |
| 2004 | 1.08 | 0.89 | 1.30 | 1.07 | 0.89 | 1.30 | |
| 2005 | 1.12 | 0.92 | 1.36 | 1.11 | 0.92 | 1.35 | |
| 2006 | 1.07 | 0.88 | 1.31 | 1.06 | 0.87 | 1.30 | |
| 2007 | 1.00 | 0.82 | 1.21 | 0.98 | 0.81 | 1.20 | |
| 2008 | 0.90 | 0.73 | 1.12 | 0.89 | 0.72 | 1.11 | |
| 2009 | 0.82 | 0.66 | 1.02 | 0.81 | 0.65 | 1.01 | |
| 2010 | 0.83 | 0.66 | 1.05 | 0.82 | 0.66 | 1.03 | |
| 2011 | 0.78 | 0.62 | 1.00 | 0.77 | 0.61 | 0.98 | |
|  |  |  |  |  |  |  | |
| *N* | 98,652 | | | 98,652 | | | |
| *PY* | 615,075 | | | 615,075 | | | |
|  |  |  |  |  |  |  | |
| Notes: Includes all adult (15+ years) members of households in the demographic surveillance. Results are from an exponential regression model, regressing time to HIV-related death on year, female, year*female, age category indicators (15-29, 30-44, 45-64, 65+) and interactions of age with female. Relative risks (hazard ratios) shown in the figure are exponentiated coefficients on year*female, i.e. the excess risk of dying from HIV for women vis-à-vis men, standardized to the 2003 relative risk. | | | | | | | |
