# Supplementary material for: Mass HIV Treatment and Sex Disparities in Life Expectancy: Demographic Surveillance in Rural South Africa
Source: PLoS Med. 2015 Nov 24;12(11):e1001905. doi: 10.1371/journal.pmed.1001905 (PMC4658174; doi:10.1371/journal.pmed.1001905)
Supplement: S3 Table — (DOCX) [file pmed.1001905.s006.docx]

**S2 Table. HIV mortality rates by age, sex, and year, 2001-2011**

|  |  | **Female** | | | **Male** | | |  |  |  |
| --- | --- | --- | --- | --- | --- | --- | --- | --- | --- | --- |
| **Age** | **Year** | **Deaths** | **PY/100** | **Rate** | **Deaths** | **PY/100** | **Rate** | **Rate Ratio** | **95% CI** | |
| 15-29 | 2001 | 149 | 135.8 | 1.10 | 67 | 128.5 | 0.52 | 2.10 | 1.58 | 2.81 |
|  | 2002 | 151 | 141.5 | 1.07 | 78 | 133.8 | 0.58 | 1.83 | 1.39 | 2.41 |
|  | 2003 | 148 | 143.1 | 1.03 | 76 | 135.8 | 0.56 | 1.85 | 1.40 | 2.44 |
|  | 2004 | 133 | 145.1 | 0.92 | 64 | 138.6 | 0.46 | 1.99 | 1.47 | 2.68 |
|  | 2005 | 121 | 146.6 | 0.83 | 55 | 141.0 | 0.39 | 2.12 | 1.54 | 2.91 |
|  | 2006 | 108 | 148.7 | 0.73 | 52 | 144.2 | 0.36 | 2.01 | 1.45 | 2.80 |
|  | 2007 | 115 | 152.9 | 0.75 | 54 | 148.5 | 0.36 | 2.07 | 1.50 | 2.86 |
|  | 2008 | 78 | 154.5 | 0.50 | 42 | 150.4 | 0.28 | 1.81 | 1.24 | 2.63 |
|  | 2009 | 65 | 155.8 | 0.42 | 39 | 152.1 | 0.26 | 1.63 | 1.09 | 2.42 |
|  | 2010 | 71 | 157.6 | 0.45 | 40 | 154.7 | 0.26 | 1.74 | 1.18 | 2.57 |
|  | 2011 | 57 | 158.5 | 0.36 | 34 | 155.4 | 0.22 | 1.64 | 1.07 | 2.51 |
| 30-44 | 2001 | 154 | 76.0 | 2.03 | 164 | 63.1 | 2.60 | 0.78 | 0.63 | 0.97 |
|  | 2002 | 186 | 78.1 | 2.38 | 204 | 64.8 | 3.15 | 0.76 | 0.62 | 0.92 |
|  | 2003 | 220 | 77.7 | 2.83 | 223 | 64.6 | 3.45 | 0.82 | 0.68 | 0.99 |
|  | 2004 | 207 | 76.8 | 2.70 | 186 | 64.1 | 2.90 | 0.93 | 0.76 | 1.13 |
|  | 2005 | 185 | 76.6 | 2.41 | 173 | 64.1 | 2.70 | 0.89 | 0.73 | 1.10 |
|  | 2006 | 152 | 77.4 | 1.96 | 147 | 65.0 | 2.26 | 0.87 | 0.69 | 1.09 |
|  | 2007 | 161 | 79.0 | 2.04 | 164 | 67.0 | 2.45 | 0.83 | 0.67 | 1.03 |
|  | 2008 | 119 | 78.0 | 1.53 | 132 | 67.0 | 1.97 | 0.77 | 0.60 | 0.99 |
|  | 2009 | 101 | 78.0 | 1.29 | 121 | 67.7 | 1.79 | 0.72 | 0.56 | 0.94 |
|  | 2010 | 85 | 79.2 | 1.07 | 108 | 69.9 | 1.54 | 0.69 | 0.52 | 0.92 |
|  | 2011 | 77 | 80.3 | 0.96 | 103 | 71.3 | 1.44 | 0.66 | 0.49 | 0.89 |
| 45-64 | 2001 | 64 | 44.4 | 1.44 | 91 | 35.6 | 2.56 | 0.56 | 0.41 | 0.78 |
|  | 2002 | 50 | 46.2 | 1.08 | 101 | 36.7 | 2.75 | 0.39 | 0.28 | 0.55 |
|  | 2003 | 75 | 47.6 | 1.58 | 97 | 36.9 | 2.63 | 0.60 | 0.44 | 0.81 |
|  | 2004 | 80 | 48.9 | 1.64 | 99 | 36.7 | 2.70 | 0.61 | 0.45 | 0.82 |
|  | 2005 | 87 | 49.1 | 1.77 | 92 | 36.7 | 2.50 | 0.71 | 0.53 | 0.95 |
|  | 2006 | 66 | 49.8 | 1.33 | 85 | 36.8 | 2.31 | 0.58 | 0.42 | 0.79 |
|  | 2007 | 65 | 50.9 | 1.28 | 91 | 37.3 | 2.44 | 0.52 | 0.38 | 0.72 |
|  | 2008 | 55 | 52.0 | 1.06 | 73 | 37.7 | 1.94 | 0.55 | 0.38 | 0.77 |
|  | 2009 | 50 | 53.2 | 0.94 | 77 | 37.9 | 2.03 | 0.46 | 0.32 | 0.66 |
|  | 2010 | 39 | 55.0 | 0.71 | 62 | 38.2 | 1.62 | 0.44 | 0.29 | 0.65 |
|  | 2011 | 35 | 55.7 | 0.63 | 60 | 38.2 | 1.57 | 0.40 | 0.26 | 0.61 |
| 65+ | 2001 | 12 | 22.1 | 0.54 | 25 | 10.4 | 2.40 | 0.23 | 0.11 | 0.45 |
|  | 2002 | 26 | 22.6 | 1.15 | 20 | 10.3 | 1.95 | 0.59 | 0.33 | 1.06 |
|  | 2003 | 22 | 22.7 | 0.97 | 27 | 10.1 | 2.67 | 0.36 | 0.21 | 0.64 |
|  | 2004 | 20 | 23.2 | 0.86 | 22 | 10.2 | 2.16 | 0.40 | 0.22 | 0.73 |
|  | 2005 | 28 | 24.3 | 1.15 | 21 | 10.4 | 2.02 | 0.57 | 0.32 | 1.00 |
|  | 2006 | 28 | 25.1 | 1.12 | 17 | 10.6 | 1.60 | 0.70 | 0.38 | 1.27 |
|  | 2007 | 28 | 25.3 | 1.11 | 30 | 10.9 | 2.76 | 0.40 | 0.24 | 0.67 |
|  | 2008 | 12 | 25.4 | 0.47 | 21 | 10.9 | 1.93 | 0.24 | 0.12 | 0.50 |
|  | 2009 | 19 | 25.0 | 0.76 | 26 | 10.7 | 2.43 | 0.31 | 0.17 | 0.56 |
|  | 2010 | 16 | 24.8 | 0.65 | 24 | 10.7 | 2.25 | 0.29 | 0.15 | 0.54 |
|  | 2011 | 9 | 24.4 | 0.37 | 13 | 10.5 | 1.24 | 0.30 | 0.13 | 0.70 |
