# Supplementary material for: Mass HIV Treatment and Sex Disparities in Life Expectancy: Demographic Surveillance in Rural South Africa
Source: PLoS Med. 2015 Nov 24;12(11):e1001905. doi: 10.1371/journal.pmed.1001905 (PMC4658174; doi:10.1371/journal.pmed.1001905)
Supplement: S4 Table — (DOCX) [file pmed.1001905.s007.docx]

**S3 Table. HIV deaths by year and type, age 15 years and over**

|  | **Female** | | | | | **Male** | | | | | |
| --- | --- | --- | --- | --- | --- | --- | --- | --- | --- | --- | --- |
| **Year** | **Never sought care** | **Pre-ART care** | **ART < 1 year** | **ART > 1 year** | **TOTAL** |  | **Never sought care** | **Pre-ART care** | **ART < 1 year** | **ART > 1 year** | **TOTAL** |
|  |  |  |  |  |  |  |  |  |  |  |  |
| 2001 | 247 | 0 | 0 | 0 | 247 |  | 225 | 0 | 0 | 0 | 225 |
| 2002 | 286 | 0 | 0 | 0 | 286 |  | 233 | 0 | 0 | 0 | 233 |
| 2003 | 311 | 0 | 0 | 0 | 311 |  | 249 | 0 | 0 | 0 | 249 |
| 2004 | 287 | 0 | 0 | 0 | 287 |  | 199 | 0 | 0 | 0 | 199 |
| 2005 | 289 | 0 | 3 | 0 | 292 |  | 202 | 0 | 5 | 0 | 207 |
| 2006 | 226 | 0 | 11 | 2 | 239 |  | 169 | 0 | 5 | 0 | 174 |
| 2007 | 173 | 35 | 29 | 6 | 243 |  | 168 | 19 | 15 | 5 | 207 |
| 2008 | 91 | 36 | 30 | 8 | 165 |  | 101 | 23 | 19 | 8 | 151 |
| 2009 | 79 | 26 | 33 | 17 | 155 |  | 104 | 19 | 26 | 18 | 167 |
| 2010 | 71 | 35 | 27 | 17 | 150 |  | 88 | 26 | 27 | 9 | 150 |
| 2011 | 47 | 20 | 25 | 26 | 118 |  | 65 | 15 | 18 | 20 | 118 |
|  |  |  |  |  |  |  |  |  |  |  |  |

HIV deaths are identified via verbal autopsy (93% response rates) and using the InterVA assignment algorithm. HIV deaths include TB-related deaths, as TB is a very common opportunistic infection for HIV. All deaths that occurring within three months of migrating into the DSA were excluded as the deceased may not have had the opportunity to seek care in the DSA.
