# Supplementary material for: Mass HIV Treatment and Sex Disparities in Life Expectancy: Demographic Surveillance in Rural South Africa
Source: PLoS Med. 2015 Nov 24;12(11):e1001905. doi: 10.1371/journal.pmed.1001905 (PMC4658174; doi:10.1371/journal.pmed.1001905)
Supplement: S5 Table — (DOCX) [file pmed.1001905.s008.docx]

**S4 Table. Proportion of HIV-deceased in 2011 who never sought care, using alternate definitions of HIV-related deaths**

| **Definition of HIV-related deaths** | **Female % (n)** | **Male % (n)** |
| --- | --- | --- |
|  |  |  |
| InterVA-assigned HIV/TB-related | 40% (47) | 55% (65) |
| HIV named as cause | 49% (22) | 58% (15) |
| HIV named as cause; likelihood > 90% | 42% (16) | 62% (13) |
| HIV or TB named as cause | 44% (56) | 58% (74) |
| HIV or TB named as cause; likelihood > 90% | 42% (49) | 57% (67) |
|  |  |  |

HIV deaths are identified via verbal autopsy (93% response rates) and using the InterVA assignment algorithm. Here we assess robustness of the main results, listed in the top row, to alternate assignment rules for HIV-related deaths. Although the total number of deaths attributed to HIV varies widely, the proportion of those deaths among persons who had never sought public sector HIV care was remarkably consistent across specifications.
