# Supplementary material for: Mass HIV Treatment and Sex Disparities in Life Expectancy: Demographic Surveillance in Rural South Africa
Source: PLoS Med. 2015 Nov 24;12(11):e1001905. doi: 10.1371/journal.pmed.1001905 (PMC4658174; doi:10.1371/journal.pmed.1001905)
Supplement: S1 Text — (DOCX) [file pmed.1001905.s009.docx]

**S1 Sensitivity Analysis**

Supporting information for:

**Unequal Benefits From HIV Treatment: A Growing Male Disadvantage in Life Expectancy in Rural South Africa**

Jacob Bor^1,2,3*^, Sydney Rosen^1,3^, Natsayi Chimbindi^2^, Noah Haber^2,4^, Kobus Herbst^2^, Tinofa Mutevedzi^2^, Frank Tanser^2^, Deenan Pillay^2,5^, Till Bärnighausen^2,4^

1. Department of Global Health, Boston University School of Public Health, Boston, USA
2. Wellcome Trust Africa Centre for Health and Population Studies, Mtubatuba, South Africa
3. Health Economics and Epidemiology Research Office, South Africa
4. Department of Global Health and Population, Harvard School of Public Health, Boston, USA
5. Faculty of Medical Sciences, University College London, London, UK

* [jbor@bu.edu](mailto:jbor@bu.edu)

**Sensitivity of HIV-cause-deleted adult life expectancy to non-independence of HIV and competing mortality risks**

Under the assumption that HIV-related and non-HIV-related mortality risks are independent, HIV-cause-deleted adult life expectancy provides a perfect counterfactual for what adult life expectancy would be if HIV mortality were eliminated. This assumption is strong cannot be tested directly: an individual can only die once, so the correlation between causes of death is unobserved at the individual level. Although the independence assumption cannot be directly tested, we can generate evidence to strengthen or weaken our belief that the assumption holds. In this Supporting Information, we provide such evidence from four sources: (1) the existing literature, (2) response of non-HIV-related mortality to an exogenous shock to HIV mortality, (3) an extreme lower bound on HIV-cause-deleted adult life expectancy, and (4) a quantitative sensitivity analysis to strong positive or negative dependence between causes of death. In all cases, we find support for this assumption and for our interpretation of HIV-cause-deleted life expectancy as a plausible counterfactual for adult life expectancy if HIV mortality were eliminated, e.g. through further gains in treatment and prevention.

*(1) Existing Literature.* Evidence from large cohort studies, both in sub-Saharan Africa[1,2] and in developed countries[3], has shown that life expectancy among HIV-infected people receiving ART is nearly the same as life expectancy of comparable HIV-uninfected populations. These findings suggest that once ART has (nearly) eliminated death due to HIV, people with HIV face mortality risks not substantially different from the general population.

*(2) Response to an Exogenous Shock.* If there were an exogenous shock to HIV-mortality, then – if causes were correlated – one would expect to see changes in mortality due to other causes. In fact, this is precisely the scenario that we study. Mass provision of ART dramatically reduced HIV mortality rates and was not expected to have affected mortality from other causes directly. If HIV and other causes of death were positively correlated, then as ART scale-up reduced HIV mortality at the population level, we would expect to see an increase in mortality rates due to other causes, and HIV-cause-deleted adult life expectancy would decline over time. On the other hand if people with HIV were less likely than others to die from other causes, then HIV-cause-deleted life expectancy would be expected to increase with ART scale-up. In fact, as we show in the main paper, HIV-cause-deleted life expectancy is quite stable over the full follow-up period, 2001-2011, consistent with the assumption that HIV and other causes of death were not correlated. This finding holds overall[4] and, as we show in this paper, separately for men and women. Of course, we cannot with logical force rule out that there were secular trends in non-HIV-related mortality, of equal magnitude and opposite direction to the selection effect, which perfectly cancelled out trends in HIV-cause-deleted life expectancy. But such a situation would be seem to be a highly unlikely coincidence.

*(3) An Extreme Lower Bound.* It is worth thinking about what one would expect to see if HIV and other causes were correlated. As an extreme lower bound, consider the case in which there is perfect correlation between HIV-related deaths and non-HIV-related deaths, such that – in the counterfactual world, if a person didn't die of HIV, she would have died from another cause on that same day. In fact, we already know what this would look like. In this extreme scenario, HIV-cause-deleted adult life expectancy would be identical to the adult life expectancy estimates in the main paper (Figure 1) in which HIV-related deaths are included. Deaths are occurring at the same time, just due to different causes. The fact that our actual estimates of HIV-cause-deleted adult life expectancy do not track the fall and rise in all-cause adult life expectancy implies that reality was far from this extreme scenario.

*(4) Sensitivity Analyses.* How strong would the correlation between HIV and non-HIV-related causes have to be to materially affect our estimates of HIV-cause-deleted adult life expectancy? In sensitivity analyses, we considered scenarios in which persons who died from HIV would have had mortality rates from other causes equal to (i) twice and (ii) half the non-HIV mortality rates of people who did not (or had not yet) died from HIV. Positive or negative dependencies of this magnitude are possible but highly unlikely given the existing literature reviewed above.

In our study, for the estimation of HIV cause-deleted life expectancy, we followed the standard demographic approaches to estimate cause-deleted life expectancy, which censor persons at date of death if they died from HIV. Such censoring is analogous to the assumption that these persons – had they not died from HIV –would have experienced the same age-specific mortality rates as the other persons left in the population who did not die from HIV. To test the sensitivity of our results to violations of this assumption, we imputed dates of death by subjecting persons who died from HIV to three potential survival curves beginning at their observed age at death: the observed survival curve (which is expected to yield a result very similar to observed HIV-cause-deleted life expectancy); a survival curve in which people who died from HIV were assumed to have twice the risk of death at every age compared to the surviving population (strong positive correlation); and a survival curve in which people who died from HIV were assumed to have half the risk of death at every age compared to the surviving population (strong negative correlation). We then calculated HIV-cause-deleted adult life expectancy for the full population, with dates of death due to other causes imputed for people who died from HIV, under the three scenarios.

We proceeded as follows. (1) We constructed a pooled (2001-2011) HIV-cause-deleted survival curve, S_baseline. (2) We constructed two additional survival curves representing strongly positive and strongly negative dependencies. S_double was created by doubling age-specific mortality rates; and S_half was created by halving age-specific mortality rates (Figure A, below). (3) For every HIV-related death identified in the surveillance by verbal autopsy (2001-2011), we randomly sampled a date of death after that date from each of the three survival curves: S_baseline, S_double, and S_half. Specifically, we drew a random number from a uniform distribution over the interval (0, S(Age at HIV death)), then inverted the survival function to obtain imputed “age at death” under each of the three scenarios, and then added the extra years lived (imputed age at death minus observed age at death plus 0.5) to the dates of death observed in the surveillance. (4) We calculated HIV-cause-deleted adult life expectancy under each of these scenarios for men and women separately, for each calendar year 2001-2011 and plotted these against observed HIV-cause-deleted life expectancy and observed all cause adult life expectancy, similar to Figure 1 in the manuscript (Figures B and C, below).

Our estimates imputing from S_baseline were very similar to our observed estimates of HIV-cause-deleted adult life expectancy, as would be expected because they were generated under the same set of assumptions, namely independence of HIV and other causes of death. Figures B and C below display HIV-cause-deleted adult life expectancy under three scenarios: observed, as reported in the main paper; in which people who died of HIV were twice as likely to die from other causes; and in which people who died from HIV were half as likely to die from other causes. These scenarios are overlaid with our estimates of all-cause adult life expectancy as reported in the main paper.

As shown in Figures B and C, these scenarios – which reflect quite extreme and unlikely levels of dependence between causes of death[1–3] – in fact provide quite tight bounds around HIV-cause-deleted adult life expectancy. These results thus strongly confirm the robustness of our findings to violations of the independence assumption. This very high degree of robustness can be understood by considering our data. Even in this high mortality population, death is still a rare event. Thus, doubling the mortality rate yields distributions of dates of death that are still very much overlapping. We interpret these sensitivity analyses as providing a plausible range for the adult life expectancy that would be observed if HIV were eliminated as a cause of death. The fact that the bounds are so tight – even for extreme scenarios – supports our interpretation of HIV-cause-deleted adult life expectancy as a valid estimate of this counterfactual state of the world.

**Figure A. Three survival curves (observed, twice-the-risk, half-the-risk) from which we sampled counterfactual dates of death for people who died from HIV**

**Figure B. Female adult LE and HIV-cause-deleted adult LE in three scenarios.**

**Figure C. Male adult LE and HIV-cause-deleted adult LE in three scenarios.**
