# Supplementary material for: Mass HIV Treatment and Sex Disparities in Life Expectancy: Demographic Surveillance in Rural South Africa
Source: PLoS Med. 2015 Nov 24;12(11):e1001905. doi: 10.1371/journal.pmed.1001905 (PMC4658174; doi:10.1371/journal.pmed.1001905)
Supplement: S2 Text — (DOCX) [file pmed.1001905.s010.docx]

**S1 Adherence to Analysis Protocol**

Supporting information for:

**Unequal Benefits From HIV Treatment: A Growing Male Disadvantage in Life Expectancy in Rural South Africa**

Jacob Bor^1,2,3*^, Sydney Rosen^1,3^, Natsayi Chimbindi^2^, Noah Haber^2,4^, Kobus Herbst^2^, Tinofa Mutevedzi^2^, Frank Tanser^2^, Deenan Pillay^2,5^, Till Bärnighausen^2,4^

1. Department of Global Health, Boston University School of Public Health, Boston, USA
2. Wellcome Trust Africa Centre for Health and Population Studies, Mtubatuba, South Africa
3. Health Economics and Epidemiology Research Office, South Africa
4. Department of Global Health and Population, Harvard School of Public Health, Boston, USA
5. Faculty of Medical Sciences, University College London, London, UK

* [jbor@bu.edu](mailto:jbor@bu.edu)

No prospective protocol was published or registered for this observational study. However we followed a clear analysis plan, as described in the methods section, and did not deviate from this plan.

- The inclusion/exclusion criteria for the study were established at the outset and were not changed. The study included all members of all households residing in a geographic surveillance area under demographic surveillance by the Africa Centre. We restricted our analyses to 2001 – 2011, years for which verbal autopsy data were available at the time when the analysis was conducted. Our analyses included the complete study population – there was no sampling.
- The statistical analyses were determined at the outset and were not changed, although we later added robustness checks. We planned to: (i) measure sex-specific changes in adult life expectancy and HIV-cause deleted life-expectancy; (ii) assess changes in age-standardized sex-specific HIV-mortality and to assess the evolution of male-female relative rates of HIV mortality with ART scale up; and (iii) to identify where in the cascade of care HIV deaths occur.
- Our methods for calculating adult life expectancy and HIV-cause deleted life expectancy at annual intervals were determined at the outset and were identical to a previous paper published in 2013 (Science), which reported aggregate (pooled-sex) trends.
- All subgroup analyses conducted were pre-specified. Assessing trends by sex was a specific aim of the paper. Further disaggregating by age enabled us to test the hypothesis that age was confounding the by-sex trends (it was not). Standard age categories were used in the analysis.
- Our definitions of the stages of HIV care were defined at the outset and were based on prior studies that we and others have published, which have used first CD4 count as the date of entry into care and have noted differential mortality patterns in the first vs. later years after initiating ART. These definitions were not changed.
- Our outcomes – death to any cause and HIV-related death – were determined at the outset. For both theoretical and logistical reasons (low response rates in HIV surveillance) we used the Africa Centre’s nearly complete verbal autopsy data.
