# Supplementary material for: Mass HIV Treatment and Sex Disparities in Life Expectancy: Demographic Surveillance in Rural South Africa
Source: PLoS Med. 2015 Nov 24;12(11):e1001905. doi: 10.1371/journal.pmed.1001905 (PMC4658174; doi:10.1371/journal.pmed.1001905)
Supplement: S3 Text — (DOC) [file pmed.1001905.s011.doc]

**S3 Text. STROBE Checklist**

Supplement to:

**Unequal Benefits From HIV Treatment: A Growing Male Disadvantage in Life Expectancy in Rural South Africa**

Jacob Bor^1,2,3*^, Sydney Rosen^1,3^, Natsayi Chimbindi^2^, Noah Haber^2,4^, Kobus Herbst^2^, Tinofa Mutevedzi^2^, Frank Tanser^2^, Deenan Pillay^2,5^, Till Bärnighausen^2,4^

1. Department of Global Health, Boston University School of Public Health, Boston, USA
2. Wellcome Trust Africa Centre for Health and Population Studies, Mtubatuba, South Africa
3. Health Economics and Epidemiology Research Office, University of Witwatersrand, Johannesburg, South Africa
4. Department of Global Health and Population, Harvard School of Public Health, Boston, USA
5. Faculty of Medical Sciences, University College London, London, UK

* Corresponding author: Jacob Bor, 801 Massachusetts Avenue, 3^rd^ Floor, Boston, MA 02118, (tel) +1 617 414 1444, (email) [jbor@bu.edu](mailto:jbor@bu.edu)

|  | Item No | Recommendation |
| --- | --- | --- |
| **Title and abstract** | 1 | (*a*) Indicate the study’s design with a commonly used term in the title or the abstract  Title: Demographic surveillance  Abstract: “population-based surveillance cohort” |
|  |  | (*b*) Provide in the abstract an informative and balanced summary of what was done and what was found  Abstract: “Methods and Findings” section |
| Introduction | | |
| Background/rationale | 2 | Explain the scientific background and rationale for the investigation being reported  Introduction: paragraphs 1, 3-5 |
| Objectives | 3 | State specific objectives, including any prespecified hypotheses  Introduction: paragraph 2: “This paper investigates the differential impact of ART scale-up on survival of men and women in the general population. We assess sex-specific trends in adult life expectancy and HIV-related mortality. To shed light on why men and women continue to die from HIV when ART is widely available, we investigate where in the cascade of care HIV mortality occurs; how these patterns differ by sex; and how they have changed over time with ART scale-up.” |
| Methods | | |
| Study design | 4 | Present key elements of study design early in the paper  Methods: Data subsection, paragraph 1: “The Wellcome Trust Africa Centre for Health and Population Studies (Africa Centre) is a large health and demographic surveillance site in rural KwaZulu-Natal, South Africa. All members of all households located in a 438 km^2^ surveillance area are followed up longitudinally on a range of demographic, health, and socioeconomic indicators through regular household surveys.” |
| Setting | 5 | Describe the setting, locations, and relevant dates, including periods of recruitment, exposure, follow-up, and data collection  Methods: Data subsection, paragraphs 2-3: “The area has an adult HIV prevalence of 29% [18] and just 34% of working age adults are employed [20]. Since 2000, the Africa Centre has conducted demographic surveillance through semi-annual household visits. Over 150,000 individuals in more than 20,000 households have been included in the surveillance; household response rates exceed 99% [21]. The surveillance population includes all members of all households both resident and non-resident household members, which is an important feature given high rates of migration in rural South Africa. Following every death in the surveillance area that is recorded by the surveillance teams, the household of the deceased is visited by a trained nurse who conducts a verbal autopsy interview to determine the probable cause of death. Information on the events leading up to the death is recorded in a standardized form; these data are fed into a computerized algorithm (InterVA); and a cause of death is probabilistically assigned [22,23]. Response rates in the verbal autopsy data are very high. During the period 2001-2011, 93.0% of all deaths had a cause of death assigned by verbal autopsy; 5.3% were missing/refused, and 1.7% were indeterminate.  In addition to the demographic surveillance, the Africa Centre maintains a clinical database for all patients in the public sector HIV and Treatment program (Hlabisa HIV Treatment and Care Programme) that has served the DSA since the national HIV treatment program began in 2004. Clinical records have been linked at the individual level with the demographic surveillance using national identification numbers, full name, age, and sex, enabling assessment of the demographic implications of public sector treatment scale-up [19,20]. Private sector utilization for ART is low in the community due to the high cost of ART outside the public sector and low levels of private insurance coverage.” |
| Participants | 6 | (*a*) *Cohort study*—Give the eligibility criteria, and the sources and methods of selection of participants. Describe methods of follow-up  Methods: “Study population and inclusion criteria” subsection: “**Study population and inclusion criteria.** The study population included all resident and non-resident adult (≥15) members of all households under surveillance, 2001-2011. Dates and causes of death were ascertained through household proxy for all members of the study population – regardless of whether they resided in the surveillance area. Person time was included from the date an individual was first observed in the surveillance or their fifteenth birthday until the date that person exited the surveillance, through death or because they ceased to be a member of a household under surveillance. For analyses that disaggregated by whether a person had sought care or initiated ART, we limited the study population to persons residing in the DSA, since non-resident household members would have been likely to seek HIV care and treatment outside the Hlabisa catchment area.” |
|  |  | (*b*) *Cohort study*—For matched studies, give matching criteria and number of exposed and unexposed  *N/A* |
| Variables | 7 | Clearly define all outcomes, exposures, predictors, potential confounders, and effect modifiers. Give diagnostic criteria, if applicable  Methods, “Data” subsection, paragraph 3-4: “For this analysis, dates of birth, death, and residency episodes were obtained for the complete population under surveillance from January 2001 through December 2011. Deaths were coded as HIV-related or not HIV-related based on the verbal autopsy interviews. Since tuberculosis (TB) is a common opportunistic infection and common immediate cause of death for people with HIV in this community, TB-related deaths were coded as HIV-related, consistent with previous analyses of these data [1,22,24,25]. The use of verbal autopsy to identify HIV-related deaths has important advantages over approaches using HIV biomarker surveillance, an alternate approach in the literature [2]: first, deaths occurring in HIV-infected persons but due to other causes (e.g. vehicle accidents, non-communicable diseases) are not attributed to HIV. Second, our approach is not vulnerable to the high rates of non-response in HIV biomarker surveillance, which is likely to be correlated with true HIV status [26].  “Into the Africa Centre’s demographic database were merged clinical data from the public sector HIV care and treatment program: date of first recorded CD4 count – a proxy for the date of entry into clinical HIV care – and date of ART initiation. We divided person time into four mutually exclusive, collectively exhaustive categories: (1) never sought care (no CD4 count); (2) sought care (CD4 count) but never initiated ART; (3) initiated ART within the previous year; (4) initiated ART more than one year ago. We distinguish between the first and later years on ART due to widely documented higher mortality in the first year on therapy [27].” |
| Data sources/ measurement | 8* | For each variable of interest, give sources of data and details of methods of assessment (measurement). Describe comparability of assessment methods if there is more than one group  Methods, “Data” subsection, paragraph 1: “Since 2000, the Africa Centre has conducted demographic surveillance through semi-annual household visits. Over 150,000 individuals in more than 20,000 households have been included in the surveillance; household response rates exceed 99% [20]. The surveillance population includes all members of all households in a 438 km^2^ demographic surveillance area (DSA); it includes both resident and non-resident household members, which is an important feature given high rates of migration in rural South Africa. Following every death in the surveillance area, the household of the deceased is visited and a trained nurse conducts a verbal autopsy interview to determine the probable cause of death. Information on the events leading up to the death is recorded in a standardized form; these data are fed into a computerized algorithm (InterVA); and a cause of death is probabilistically assigned [21,22].” |
| Bias | 9 | Describe any efforts to address potential sources of bias  Methods, Data analysis, Sex-specific trends in adult life expectancy, paragraph 3: “Under the assumption that mortality due to HIV and to other causes are independent, HIV-cause-deleted life expectancy reflects the life expectancy that would exist if HIV-related mortality were eliminated. Under this assumption, the difference between HIV-cause-deleted adult life expectancy and adult life expectancy would reflect the years of life lost due to HIV. Although the independence assumption cannot be tested directly, it is consistent with prior evidence HIV-infected persons on ART in sub-Saharan Africa have life expectancies close to those in the general population[27,28]. Sensitivity analyses assessing the robustness of our HIV-cause-deleted life expectancy estimates to deviations from the independence assumption – as well as further discussion of this assumption – are presented in S1 Text: Sensitivity Analysis.”  Methods, Data analysis, Sex-specific trends in HIV-related mortality, paragraph 1: “To control for potential confounding by age, we additionally controlled for age group indicators (15-29, 30-44, 45-64, 65+ years) in an age-adjusted hazard model.”  Methods, Data analysis, Sex-specific differences in progression through the HIV cascade of care and attribution of HIV-related deaths across the cascade, paragraph 3: “The validity of our attribution of HIV-related deaths across the cascade of care depends on the accuracy of verbal autopsy coding. Persons who died from other causes but whose death was attributed to HIV might not have even been HIV positive and would have been less likely to have sought care for HIV since they would have had no reason to do so. In robustness checks, we limited the sample of deaths to those that specifically named HIV as a cause; those that named HIV as a cause with an InterVA likelihood score > 90%; those naming either HIV or TB as a cause; and those naming either HIV or TB as a cause with > 90% likelihood.” |
| Study size | 10 | Explain how the study size was arrived at  Methods, Study population and inclusion criteria, paragraph 1: “The study population included all resident and non-resident adult (≥15) members of all households under surveillance, 2001-2011.” |
| Quantitative variables | 11 | Explain how quantitative variables were handled in the analyses. If applicable, describe which groupings were chosen and why  Methods, Data Analysis, Sex-specific trends in adult life expectancy, paragraph 2: “Adult life expectancy is the number of additional years of life expected, conditional on survival to age 15, and is commonly denoted e_15_. We calculated e_15_ separately for men and women using a continuous-time approach: for each calendar year, 2001-2011, we estimated sex specific survival curves beginning at age 15 years, using the non-parametric Kaplan-Meier estimator. To obtain annual, sex-specific estimates of e_15_ we numerically integrated under the annual, sex-specific survival curves. Due to sparseness of data beyond age 95, we censored the annual survival curves at age 95 and calculated the expected number of years lived in the 80-year interval between ages 15 and 95 (_80_e_15_). A correction factor equal to e_95_ was estimated separately for each sex for the entire period 2001-2011 and was added to our annual estimates of _80_e_15_ to obtain annual estimates of e_15_. Throughout this paper, we report adult life expectancy as e_15_ + 15, so that estimates correspond to ages at death rather than years remaining at age 15 [1].” |
| Statistical methods | 12 | (*a*) Describe all statistical methods, including those used to control for confounding  Methods, Data Analysis. |
|  |  | (*b*) Describe any methods used to examine subgroups and interactions  Methods, Data Analysis, Sex-specific trends in adult life expectancy, paragraph 2: “For each year, 2001-2011, we estimated sex specific survival curves beginning at age 15 years, using the Kaplan-Meier estimator.”  Methods, Data Analysis, Sex-specific trends in HIV-related mortality, paragraph 1: “We assessed trends in HIV-specific mortality rates by sex and estimated trends in female-to-male HIV mortality rate ratios. If ART scale-up had the same proportional effect on HIV mortality for men and women, then the ratio of HIV mortality for women to HIV mortality for men would be expected to be constant over time. We estimated female-to-male HIV mortality rate ratios for each calendar year, 2001-2011, using an exponential hazard model, in which time to HIV death was regressed on indicators for each calendar year and the interaction of those indicators with sex. To test whether the relative change in HIV mortality over time differed for men and women, we estimated a similar hazard model, but included a main effect for female, set calendar year=2003 as the reference category, and made inferences on the interaction between female and calendar year=2011. To control for potential confounding by age, we additionally controlled for age group indicators (15-29, 30-44, 45-64, 65+ years) in an age-adjusted hazard model. Finally, we stratified the analysis by age. We regressed time to HIV death on indicators for year-by-age strata and indicators for sex-by-year-by-age strata. Annual age-specific female-to-male HIV mortality rate ratios were obtained by exponentiating the coefficients on the latter terms, and we tested the null hypothesis that the relative changes in HIV mortality for women vs. men over the period 2003-2011 were constant across age groups. In all models, person time was censored at exit from the surveillance, either because a person ceased to be a member of a household under surveillance or because he or she died from another cause. To adjust for non-independence of the episodes belonging to the sample individuals over time, we clustered standard errors at the individual level in all regression analyses.”  Methods, Data Analysis, Sex-specific differences in progression through the HIV cascade of care and attribution of HIV-related deaths across the cascade, paragraph 1:  “To assess sex differences in progression through the HIV cascade of care, we calculated the proportion of all surviving adults (ages 15 years and over) in the population residing in the DSA who (1) had ever sought care in the ART program as evidenced by a CD4 count or (2) had ever initiated ART in the public sector ART program. Proportions were assessed annually 2001-2011 at mid-year (July 1).”  Methods, Data Analysis, Sex-specific differences in progression through the HIV cascade of care and attribution of HIV-related deaths across the cascade, paragraph 2: “We disaggregated HIV-related deaths into four groups defined by whether the deceased had ever sought care and/or initiated ART in the public sector ART program: (1) never sought care; (2) sought care but never initiated ART; (3) initiated ART less than one year prior to death; (4) initiated ART more than one year prior to death….For each year from 2001-2011, the distribution of deaths across these four care-seeking categories was evaluated separately for men and women.” |
|  |  | (*c*) Explain how missing data were addressed  Methods, Data, paragraph 1: In the demographic surveillance, “household response rates exceed 99% [20].” Therefore we used dates of death as reported and did not impute any dates of death. |
|  |  | (*d*) *Cohort study*—If applicable, explain how loss to follow-up was addressed  Loss-to-follow-up from the demographic surveillance occurs only when a person ceases to be a member of a household under surveillance.  Methods, Study population and inclusion criteria: “Person time was included from the date an individual was first observed in the surveillance or their fifteenth birthday until the date that person exited the surveillance, through death or because they ceased to be a member of a household under surveillance.”  Methods, Data Analysis, Sex-specific trends in HIV-related mortality, paragraph 1: “In all models, person time was censored at exit from the surveillance, either because a person ceased to be a member of a household under surveillance or because he or she died from another cause.”  Results, paragraph 1: “Attrition from the population surveillance was comparable for men and women at 3.0 and 3.3 per 100 person-years, respectively.” |
|  |  | (*e*) Describe any sensitivity analyses  Methods, Data analysis, Sex-specific trends in adult life expectancy, paragraph 1: “Under the assumption that mortality due to HIV and to other causes are independent, HIV-cause-deleted life expectancy reflects the life expectancy that would exist if HIV-related mortality were eliminated. Under this assumption, the difference between HIV-cause-deleted adult life expectancy and adult life expectancy would reflect the years of life lost due to HIV. Although the independence assumption cannot be tested directly, it is consistent with prior evidence HIV-infected persons on ART in sub-Saharan Africa have life expectancies close to those in the general population[27,28]. Sensitivity analyses assessing the robustness of our HIV-cause-deleted life expectancy estimates to deviations from the independence assumption – as well as further discussion of this assumption – are presented in a Supporting Information file.”  See Supporting Information file.  Methods, Data analysis, Sex-specific differences in progression through the HIV cascade of care and attribution of HIV-related deaths across the cascade, paragraph 3:  “The validity of our attribution of HIV-related deaths across the cascade of care depends on the accuracy of verbal autopsy coding. Persons who died from other causes but whose death was attributed to HIV might not have even been HIV positive and would have been less likely to have sought care for HIV since they would have had no reason to do so. In robustness checks, we limited the sample of deaths to those that specifically named HIV as a cause; those that named HIV as a cause with an InterVA likelihood score > 90%; those naming either HIV or TB as a cause; and those naming either HIV or TB as a cause with > 90% likelihood.” |

| Results | | |
| --- | --- | --- |
| Participants | 13* | (a) Report numbers of individuals at each stage of study—eg numbers potentially eligible, examined for eligibility, confirmed eligible, included in the study, completing follow-up, and analysed  Methods, Study population and inclusion criteria, paragraph 1: “The study population included all resident and non-resident adult (≥15) members of all households under surveillance, 2001-2011.”  Results, paragraph 1: “All adult person-time contributed by members of the demographic surveillance from 2001 through 2011 was analyzed. 52,964 women and 45,688 men contributed a total of 615,075 person-years to the analysis (Table 1). Survey attrition rates were comparable for men and women at 3.0% and 3.3% per year, respectively. 12,290 deaths were reported during follow-up of which 7,229 (58.8%) were identified by verbal autopsy to be HIV/TB-related.” |
|  |  | (b) Give reasons for non-participation at each stage  Results, paragraph 1: All adult person-time contributed by members of the demographic surveillance from 2001 through 2011 was analyzed. |
|  |  | (c) Consider use of a flow diagram  All adult person time contributed by members of this population surveillance cohort from 2001 through 2011 was analysed. Therefore no participation flow diagram is presented. |
| Descriptive data | 14* | (a) Give characteristics of study participants (eg demographic, clinical, social) and information on exposures and potential confounders |
|  |  | (b) Indicate number of participants with missing data for each variable of interest |
|  |  | (c) *Cohort study*—Summarise follow-up time (eg, average and total amount)  See: **Table 1, Population demographic surveillance 2001-2011, summary statistics.** |
| Outcome data | 15* | *Cohort study*—Report numbers of outcome events or summary measures over time.  Nearly all results are reported over time:  [**Fig 1. Adult life expectancy and HIV-cause-deleted adult life expectancy, 2001-2011, by sex.** Solid markers are annual estimates of adult life expectancy; hollow markers are annual estimates of HIV-cause-deleted adult life expectancy. 95% CIs shown.]  [**Fig 2. Female – male difference in adult life expectancy, 2001-2011.** Solid blue line displays annual estimates of the gap between female and male adult life expectancy. The red dashed line displays the gap in HIV-cause-deleted life expectancy observed in 2011.]  **[Fig 3. Sex-specific survival curves: 2003, 2011, and HIV-cause-deleted.** Sex-specific continuous-time Kaplan-Meier survival curves. HIV-cause-deleted survival curve pools person time for 2001-2011.]  **Table 2, HIV mortality rates for females and males, 2001-2011**  [**Fig 4. Female – male HIV mortality rate ratios by age and year, 2004-2011**  Age-specific HIV mortality rate ratios for women vs. men were estimated in a hazard regression model which included year indicators for each age group and interactions for each age and year with sex. The pooled estimate is from a separate regression model. Mortality rate ratios declined over time in all age groups.]  [**S3 Table. HIV mortality rates by age, sex, and year, 2001-2011**]  [**Fig 5. Distribution of HIV deaths across cascade of care, 2001-2011.** We have excluded all deaths that occurred less than three months after migration into to the DSA.] |
|  |  | *Case-control study—*Report numbers in each exposure category, or summary measures of exposure  N/A |
|  |  | *Cross-sectional study—*Report numbers of outcome events or summary measures  N/A |
| Main results | 16 | (*a*) Give unadjusted estimates and, if applicable, confounder-adjusted estimates and their precision (eg, 95% confidence interval). Make clear which confounders were adjusted for and why they were included  [**Fig 1. Adult life expectancy and HIV-cause-deleted adult life expectancy, 2001-2011, by sex.** Solid markers are annual estimates of adult life expectancy; hollow markers are annual estimates of HIV-cause-deleted adult life expectancy. 95% CIs shown.]  **Table 2, HIV mortality rates for females and males, 2001-2011**  [**Fig 4. Female – male HIV mortality rate ratios by age and year, 2004-2011**  Age-specific HIV mortality rate ratios for women vs. men were estimated in a hazard regression model which included year indicators for each age group and interactions for each age and year with sex. The pooled estimate is from a separate regression model. Mortality rate ratios declined over time in all age groups.]  [**S3 Table. HIV mortality rates by age, sex, and year, 2001-2011**]  As this was a descriptive analysis, no confounders other than age were adjusted for. However, we present estimates stratified by age and sex. |
|  |  | (*b*) Report category boundaries when continuous variables were categorized  Methods, p12, paragraph 1: “To control for potential confounding by age, we additionally controlled for age group indicators (15-29, 30-44, 45-64, 65+ years) in an age-adjusted hazard model.”  Results:  **[Fig 4. Female – male HIV mortality rate ratios by age and year, 2004-2011**  Age-specific HIV mortality rate ratios for women vs. men were estimated in a hazard regression model which included year indicators for each age group and interactions for each age and year with sex. The pooled estimate is from a separate regression model. Mortality rate ratios declined over time in all age groups.]  **S3 Table. HIV mortality rates by age, sex, and year, 2001-2011** |
|  |  | (*c*) If relevant, consider translating estimates of relative risk into absolute risk for a meaningful time period  We present both relative risks of HIV mortality and absolute differences in adult life expectancy. |
| Other analyses | 17 | Report other analyses done—eg analyses of subgroups and interactions, and sensitivity analyses  **S1 Fig. Global Trends in Adult Life Expectancy and the Female-Male Adult Life Expectancy Gap, 1990 – 2000**. Source: [5].  **S3 Table. HIV mortality rates by age, sex, and year, 2001-2011**  **S2 Fig. Proportion of male and female DSA residents ages 15 years and over who have sought care for HIV (left) and/or initiated ART (right) in the public sector treatment program.** Proportion of DSA residents ages 15 and over who have ever sought care for HIV or initiated ART. In 2011, 9% of all women had initiated ART and an additional 8% had sought care and had a CD4 count but had not yet initiated ART. Just 4% of all men had initiated ART; an additional 3% had sought care but not yet initiated ART.  **Fig 5. Distribution of HIV deaths across cascade of care, 2001-2011.** Note: We have excluded all deaths that occurred less than three months after migration into to the DSA.  **S3 Fig. Distribution of HIV deaths across cascade of care, 2011.**  **S4 Table. HIV deaths by year and type, age 15 years and over**  **S5 Table. Proportion of HIV-deceased in 2011 who never sought care, using alternate definitions of HIV-related deaths**  **Table 3. HIV Mortality Rates (per 100 PY) and population shares; ages 15+** |
| Discussion | | |
| Key results | 18 | Summarise key results with reference to study objectives  Discussion, paragraph 1: “Mass provision of free ART in public sector facilities in South Africa has coincided with dramatic reductions in HIV-related mortality for both men and women. However, the decline in HIV mortality for women has substantially outpaced the decline for men. Prior to ART scale-up, epidemic HIV had substantially compressed the female-male gap in adult life expectancy, due to women’s younger average age at infection and longer lifespan in the absence of HIV. Access to life-prolonging treatment has led not only to the natural decompression of the female-male adult life expectancy gap, but also to the unanticipated emergence of male sex as a significant predictive risk factor for HIV mortality at the population level in this setting. Since the start of the ART program in 2004, the female-male gap in adult life expectancy has nearly doubled.” |
| Limitations | 19 | Discuss limitations of the study, taking into account sources of potential bias or imprecision. Discuss both direction and magnitude of any potential bias  Discussion, paragraphs 7-10: Our study had some limitations. First, due to the nature of the data, we report on only one rural setting in one province in South Africa. We note however that this area is in one of the poorest districts in South Africa and has many features – high unemployment, high migration, complex household arrangements, low private sector utilization for HIV treatment – common to rural areas with high HIV prevalence in southern Africa. Second, we report on trends in adult life expectancy and HIV mortality before and during the scale-up of public sector HIV treatment in South Africa. Although the changes in mortality patterns observed coincided with the rollout of ART in 2004 and progressive expansion of services through 2011, we cannot rule out that other contemporaneous factors could have influenced population mortality trends, such as survivorship effects. In previous analysis, we simulated the counterfactual path of the epidemic and demonstrated that no rapid increase in life expectancy was predicted due to internal dynamics of the epidemic in the absence of ART scale-up [1]. Third, our data on the cascade of HIV treatment services exclude patients seeking care in the private sector. Though a limitation, we note that private sector care-seeking for HIV treatment is rare in this setting, given the free provision of ART in the public sector. Fourth, we divided the treatment cascade into four discrete, easily defined stages: never had a CD4 count; had a CD4 count but never initiated; initiated less than a year ago; initiated more than a year ago. These definitions may be conservative: persons who sought care or initiated ART may not still be in care. Investigating sex-specific patterns of clinical attrition and churning, and their contribution to survival disparities between men and women on ART is an important topic for future research.  As with all verbal autopsy approaches, our method of identifying HIV-related deaths may have resulted in some misclassification. The quality of verbal autopsy data depends on the skill of the interviewer, the willingness of household members to participate, the accuracy of their recall, and the validity of the algorithm used to make diagnoses based on reported symptoms. The underlying data for verbal autopsy at Africa Centre come from high frequency (twice- and later thrice-annual) demographic surveillance, with autopsy interviews conducted by trained nurses on average six months following a recorded death; in contrast, other surveys have used lay health workers or field interview staff to conduct verbal autopsies and/or relied on longer recall intervals following a death [24,25]. Response rates were very high, with 93% of all deaths assigned a cause of death by verbal autopsy. An active literature has sought to develop and validate verbal autopsy classification algorithms [22,25,43,44]. We used an algorithm (InterVA), which is widely used and has been shown to have high validity in identifying HIV/TB-related deaths in populations with high HIV prevalence [22,24,25]. In particular, the algorithm has been validated locally against physician-coded verbal autopsies in the Africa Centre’s demographic surveillance [22]. TB is a common opportunistic infection in HIV-infected persons and it can be difficult to distinguish a TB-related death from an HIV-related death. Following previous studies [1,22,25], we coded all TB-related deaths as HIV-related. We note as a limitation that the inclusion of TB-related deaths may somewhat overstate the total number of HIV-related deaths, but this effect is likely to be small: a prior analysis of the Africa Centre surveillance calculated that out of the 53.5% of all adult deaths due to HIV or TB, just 5.3% were attributable to TB without HIV [45]. Further, the inclusion of TB-related mortality is appropriate given the potential spillover effect that ART scale-up may have in reducing the population burden of active TB and transmission of TB to HIV-uninfected persons [46]. Our results were robust to a variety of alternate codings of HIV-related deaths based on the InterVA algorithm.  As a final limitation, we note that our estimates of HIV-cause-deleted life expectancy would only be interpretable as counterfactual life expectancy in a world without HIV mortality if HIV and non-HIV causes of death were indeed independent. Although we cannot test this formally (individuals can only die once), we find supporting evidence from several sources: the near-normal life expectancy of patients on ART in previous studies suggests that these patients do not face substantially higher or lower mortality rates than the general population. The stability of our observed trends in HIV-cause-deleted life expectancy during a period of rapidly falling HIV-mortality is also consistent with such an interpretation. Finally, in sensitivity analyses, we found that even very large violations from the independence assumption would not substantially affect our inferences about HIV-cause-deleted life expectancy. Though we cannot reject dependence across causes, our estimates of HIV-cause-deleted life expectancy likely provide a reasonable estimate for the potential adult life expectancy that would be observed with the elimination of HIV mortality, e.g. through advances in treatment and prevention (see S1 Text: Sensitivity Analysis for further discussion of these points). |
| Interpretation | 20 | Give a cautious overall interpretation of results considering objectives, limitations, multiplicity of analyses, results from similar studies, and other relevant evidence  Discussion, paragraph 11: “Though adult life expectancy has increased dramatically for women during the scale-up of ART, the gains for men have been more modest and male sex has emerged as a risk factor for HIV mortality in this setting.” |
| Generalisability | 21 | Discuss the generalisability (external validity) of the study results  Discussion, paragraph 7: “due to the nature of the data, we report on only one rural setting in one province in South Africa. We note however that this area is in one of the poorest districts in South Africa and has many features – high unemployment, high migration, complex household arrangements, low private sector utilization for HIV treatment – common to rural areas with high HIV prevalence in southern Africa.” |
| Other information | | |
| Funding | 22 | Give the source of funding and the role of the funders for the present study and, if applicable, for the original study on which the present article is based  Funding sources are listed in the manuscript. |

*Give information separately for cases and controls in case-control studies and, if applicable, for exposed and unexposed groups in cohort and cross-sectional studies.

**Note:** An Explanation and Elaboration article discusses each checklist item and gives methodological background and published examples of transparent reporting. The STROBE checklist is best used in conjunction with this article (freely available on the Web sites of PLoS Medicine at http://www.plosmedicine.org/, Annals of Internal Medicine at http://www.annals.org/, and Epidemiology at http://www.epidem.com/). Information on the STROBE Initiative is available at [www.strobe-statement.org](http://www.strobe-statement.org/).
